# Mitochondrial Oxidative Stress Alters a Pathway in *Caenorhabditis elegans* Strongly Resembling That of Bile Acid Biosynthesis and Secretion in Vertebrates

**DOI:** 10.1371/journal.pgen.1002553

**Published:** 2012-03-15

**Authors:** Ju-Ling Liu, David Desjardins, Robyn Branicky, Luis B. Agellon, Siegfried Hekimi

**Affiliations:** 1 Department of Biology, McGill University, Montreal, Canada; 2 School of Dietetics and Human Nutrition, McGill University, Ste. Anne de Bellevue, Canada; University of California San Francisco, United States of America

## Abstract

Mammalian bile acids (BAs) are oxidized metabolites of cholesterol whose amphiphilic properties serve in lipid and cholesterol uptake. BAs also act as hormone-like substances that regulate metabolism. The *Caenorhabditis elegans clk-1* mutants sustain elevated mitochondrial oxidative stress and display a slow defecation phenotype that is sensitive to the level of dietary cholesterol. We found that: 1) The defecation phenotype of *clk-1* mutants is suppressed by mutations in *tat-2* identified in a previous unbiased screen for suppressors of *clk-1*. TAT-2 is homologous to ATP8B1, a flippase required for normal BA secretion in mammals. 2) The phenotype is suppressed by cholestyramine, a resin that binds BAs. 3) The phenotype is suppressed by the knock-down of *C. elegans* homologues of BA–biosynthetic enzymes. 4) The phenotype is enhanced by treatment with BAs. 5) Lipid extracts from *C. elegans* contain an activity that mimics the effect of BAs on *clk-1*, and the activity is more abundant in *clk-1* extracts. 6) *clk-1* and *clk-1;tat-2* double mutants show altered cholesterol content. 7) The *clk-1* phenotype is enhanced by high dietary cholesterol and this requires TAT-2. 8) Suppression of *clk-1* by *tat-2* is rescued by BAs, and this requires dietary cholesterol. 9) The *clk-1* phenotype, including the level of activity in lipid extracts, is suppressed by antioxidants and enhanced by depletion of mitochondrial superoxide dismutases. These observations suggest that *C. elegans* synthesizes and secretes molecules with properties and functions resembling those of BAs. These molecules act in cholesterol uptake, and their level of synthesis is up-regulated by mitochondrial oxidative stress. Future investigations should reveal whether these molecules are in fact BAs, which would suggest the unexplored possibility that the elevated oxidative stress that characterizes the metabolic syndrome might participate in disease processes by affecting the regulation of metabolism by BAs.

## Author Summary

Cholesterol metabolism, in particular the transport of cholesterol in the blood by lipoproteins, is an important determinant of human cardiovascular health. Bile acids are breakdown products of cholesterol that have detergent properties and are secreted into the gut by the liver. Bile acids carry out three distinct roles in cholesterol metabolism: 1) Their synthesis from cholesterol participates in cholesterol elimination. 2) They act as detergents in the uptake of dietary cholesterol from the gut. 3) They regulate many aspects of metabolism, including cholesterol metabolism, by molecular mechanisms similar to that of steroid hormones. We have found that cholesterol uptake and lipoprotein metabolism in the nematode *Caenorhabditis elegans* are regulated by molecules whose activities, biosynthesis, and secretion strongly resemble that of bile acids and which might be bile acids. Most importantly we have found that oxidative stress upsets the regulation of the synthesis of these molecules. The metabolic syndrome is a set of cardiovascular risk factors that include obesity, high blood cholesterol, hypertension, and insulin resistance. Given the function of bile acids as metabolic regulators, our findings with *C. elegans* suggest the unexplored possibility that the elevated oxidative stress that characterizes the metabolic syndrome may participate in mammalian disease processes by affecting the regulation of bile acid synthesis.

## Introduction

In mammals, cholesterol is necessary for the structure and function of membranes, and is the substrate for the biosynthesis of signalling molecules such as sexual steroids, bioactive compounds such as vitamin D, and bile acids (BAs) [1]. Cholesterol is converted into BAs through a series of oxidation reactions, as well as a shortening of the side chain in mammals (Figure S1). The enzymes that catalyze the individual biosynthetic steps of BA synthesis are localized in different cellular compartments, including the endoplasmic reticulum, cytosol, mitochondria, and peroxisomes. For example, the oxidation of the side-chain takes place in the mitochondria, but side-chain shortening takes place in the peroxisomes. In vertebrates, these reactions occur predominantly in hepatocytes.

BAs regulate cholesterol and lipid metabolism in a variety of ways. They participate in cholesterol, lipid and hydrophobic vitamin uptake through their properties as detergents. They also participate in cholesterol elimination as they are secreted into the gut from where a fraction is lost every day in the feces. However, most of the secreted BAs are taken up again through the gut epithelium and can be re-circulated to the liver and re-secreted into bile, a process that is called the entero-hepatic circulation of BAs. In addition, BAs are signalling molecules that integrate several aspects of metabolism, including fat, glucose, and energy metabolism by regulating gene expression through nuclear hormone receptors such as the farnesoid X receptor (FXR), the pregnane X receptor (PXR), and the vitamin D receptor (VDR) (BA biology is reviewed in detail in [2], [3]).

In mammals, BA excretion and recirculation depend on a number of membrane transporters such as ATP8B1 and ABCB11. ATP8B1, a type 4 P-type ATPase is a predicted phospholipid flippase [4]. Flippases transfer lipids from one leaflet of the membrane to the other thus changing the composition of both leaflets and the properties of the membranes. Several studies in mice suggest that ATP8B1 deficiency causes loss of canalicular membrane phospholipid asymmetry and as a result the resistance of the canalicular membrane to hydrophobic BAs is decreased, which impairs the activity of ABCB11, the BA export pump, and causes cholestasis, a pathological retention of bile [5]. Mutation of ATP8B1 in humans leads to progressive familial intrahepatic cholestasis type 1 (PFIC1) [6].

ATP8B1 shares 56% sequence identity with *C. elegans* TAT-2 (for Transbilayer Amphipath Transporters) [4], [7], [8]. A *tat-2* mutant was found to exhibit hypersensitivity to low dietary cholesterol with decreased reproductive growth [8]. *tat-2* mutation also suppresses the conditional growth arrest phenotypes resulting from mutation of *elo-5*, a gene encoding a very long chain fatty acid (VLCFA) elongase, which is required for the production of two monomethyl branched-chain fatty acids (mmBCFAs) in *C. elegans* [7]. As *tat-2* also partially suppresses developmental defects caused by reduction of the expression of *sptl-1*, which disrupts sphingolipid biosynthesis, the authors proposed that TAT-2 acts by affecting the localization of mmBCFA-containing sphingolipids.

Like vertebrates, *C. elegans* need sterols (reviewed in [9]). However, as *C. elegans* is capable only of modifying sterols and not of synthesising them de novo, worms are auxotrophic for sterols, which have to be added to the culture media (generally at 5 µg/ml cholesterol). A reduction in sterol supplementation leads to a complex phenotype that includes abnormal moulting, and inappropriate dauer formation. A complete lack of sterol supplementation leads to lethality. As sterols appear to be required only in very small amounts for normal physiology in worms, the deficit resulting from the absence of dietary cholesterol might result from deficits in the synthesis of signalling molecules derived from cholesterol. Indeed BA-like molecules derived from cholesterol have been identified in *C. elegans* and shown to have roles in signalling [10]. Dafachronic acid, which is required for bypassing dauer formation, has some characteristics of BAs, with oxidation of the steroid ring and of the side-chain, but its oxidation is not extensive and the side-chain is not shortened [10]. Yet, like vertebrate steroids and BAs, it acts via a nuclear hormone receptor, encoded by *daf-12* [10].

In mammals, after BA-mediated absorption, ingested lipids, cholesterol, and lipid-soluble vitamins, are transported from the gut to the tissues that need them via circulating lipoproteins such as chylomicrons. Other lipoproteins such as low density lipoproteins (LDL) distribute lipids and cholesterol from the liver to peripheral tissues, and high density lipoproteins (HDL) transport cholesterol from peripheral tissues back to the liver in a process termed reverse cholesterol transport. The best known lipoproteins in *C. elegans* are the yolk particles. The protein moieties of yolk particles are vitellogenins, distant homologues of ApoB, which is the apolipoprotein in chylomicrons and LDL [11]. In *C. elegans*, cholesterol, fatty acids, and possibly other nutrients are transported from the gut to developing oocytes through the pseudocoelomic cavity by means of yolk particles [12], [13]. However, several observations suggest that there is another lipid transport system in worms [14]. For example, hermaphrodites are capable of transporting cholesterol before the vitellogenins are expressed and males do not express vitellogenins yet accumulate cholesterol in developing sperm [13]. Furthermore, a mutation in *dsc-4*, which encodes the worm homologue of the microsomal triglyceride transfer protein (MTP) [15], which is required in mammals for the synthesis of LDL in the ER, produces multiple phenotypic effects without affecting yolk production.

CLK-1 is a conserved mitochondrial enzyme that is necessary for the biosynthesis of the antioxidant and redox cofactor ubiquinone (co-enzyme Q; CoQ). Mutations in *C. elegans clk-1* or its mouse orthologue affect mitochondrial function [16], [17], in particular they increase mitochondrial oxidative stress in both organisms [18], [19]. In worms, this results in a number of phenotypes, in particular slow development, slow aging, and slow rhythmic behaviours such as defecation [20].

The defecation cycle of *C. elegans* generates rhythmic body muscle contractions. This is a well-studied, highly regulated behaviour that is readily quantifiable [21]. *dsc-4/mtp* was originally identified as a mutation that suppresses the slow defecation of *clk-1* mutants [22]. Given the known function of MTP it was concluded that a type of MTP-dependent, LDL-like lipoprotein, distinct from yolk, affects the rate of defecation [14]. Reducing the level of dietary cholesterol mimics the effects of *dsc-4* on the defecation cycle length of *clk-1* mutants [15], [23]. These observations suggest that *clk-1* mutants have slow defecation because they have high levels of LDL-like lipoproteins biosynthesis and secretion. Furthermore, the MTP-dependent lipid transport system appears to be so well conserved between mammals and *C. elegans* that drugs that have been developed to lower lipid levels in humans can act as suppressors of the slow defecation rate of *clk-1* [23]. In particular, the slow defecation is suppressed by drugs that antagonize high LDL levels by increasing HDL levels (e.g. an inhibitor of the HDL receptor SR-BI [24]), or that reverse cholesterol transport by stimulating gene expression through nuclear hormone receptors (e.g. gemfibrozil [25]). Thus, although it is not yet known how elevated lipoprotein biosynthesis slows down the defecation cycle, the *clk-1* mutants provide a tractable genetic model for characterizing the mechanisms of lipids and sterol uptake and the biosynthesis and secretion of LDL-like lipoproteins.

Here, using genetic and pharmacological approaches, we show that sterol uptake in *C. elegans* depends on molecules that are functionally similar to BAs and might be structurally similar as well. These molecules are distinct from dafachronic acids and are synthesized and secreted through a pathway that appears to be molecularly very similar to that of BA synthesis and secretion in mammals. We also show that this pathway is altered by the high mitochondrial oxidative stress of *clk-1* mutants. A link of oxidative stress and aging with dyslipidemia and with the other cardiovascular risk factors that constitute the metabolic syndrome has repeatedly been evidenced in mammals, but its mechanistic basis has not yet been elucidated. Our findings suggest that the link could be a perturbation of BA biosynthesis, a possibility that has not yet been explored in mammals.

## Results

### The defecation phenotype of *clk-1* is suppressed by *tat-2*

**Figure 1:**
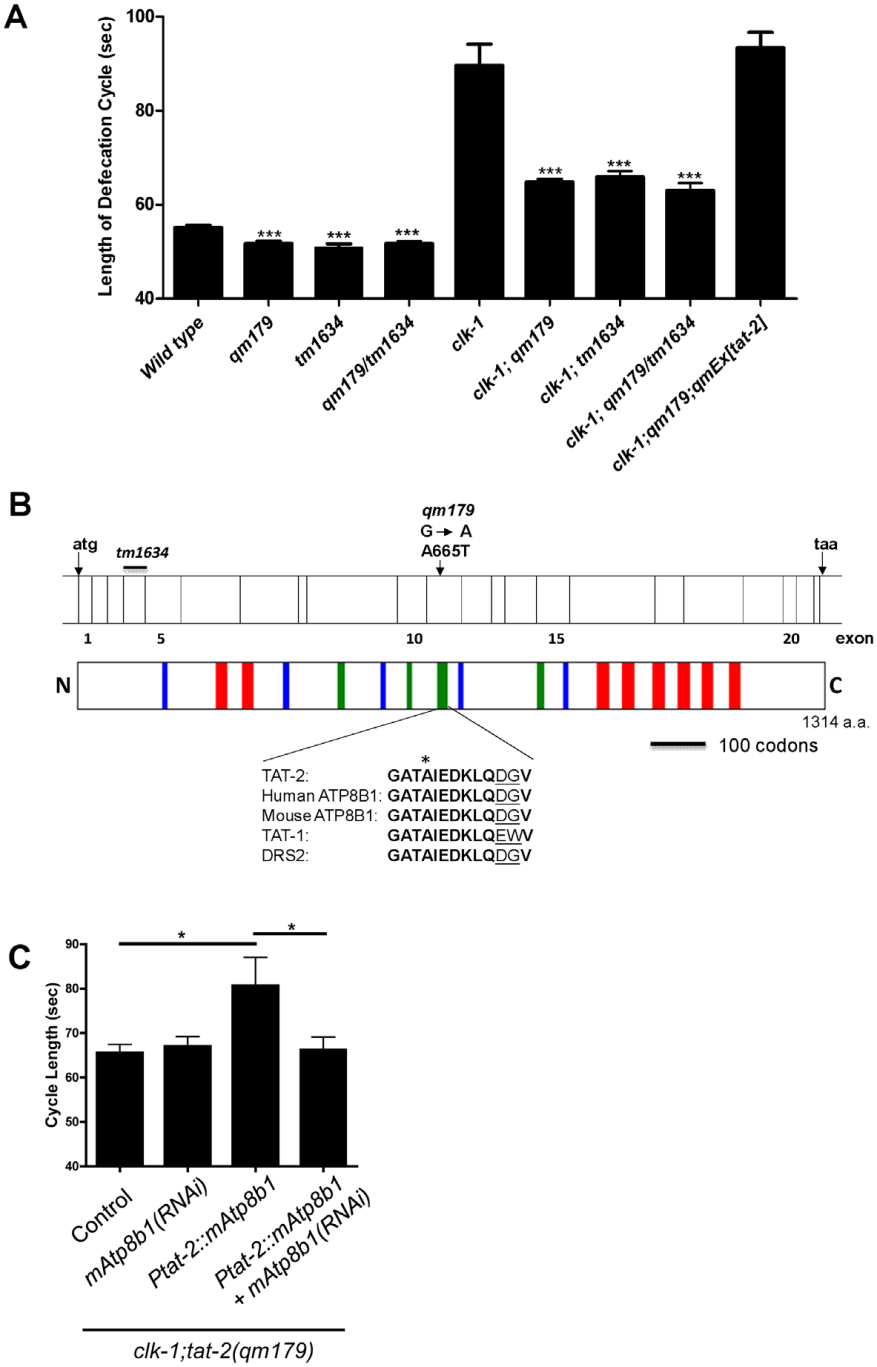
*qm179* is allelic to *tat-2*, which encodes a protein homologous to mammalian ATP8B1. (A) Both the *qm179* and *tat-2(tm1634)* deletion mutation show a short defecation cycle in the wild type background and suppress the slow defecation of *clk-1(qm30)* mutants in double mutant combinations. Furthermore the *tat-2(tm1634)* deletion mutations fails to complement *qm179* in both the wild type and *clk-1* backgrounds. The bars represent the mean defecation cycle of animals that have been scored for three consecutive defecation cycles each in the case of *clk-1(qm30)* mutants and for five consecutive defecation cycles for all other genotypes. The error bars represent S.E.M. (n≥20 animals for each genotype). The asterisks indicate that the data are significantly different from that of wild-type or *clk-1* mutants. All differences were significant at P<0.0001 by a t-test. (B) Schematic representation of the *tat-2* coding region and the lesions of the two alleles. TAT-2 is a membrane protein with 8 predicted transmembrane domains (red) and several consensus domains for all classes of P-type ATPases (blue) or specific for the P4 P-type ATPase (green). One of the P4 P-type ATPase-specific consensus domains harbors the residue that is changed in the *qm179* allele (alanine 665 to threonine). This residue (indicated by an asterisk) is absolutely conserved among mammalian ATP8B1, *C. elegans* TAT-1, and yeast DRS2. Residues that are not perfectly conserved in this region are underlined. (C) The suppression of *clk-1* produced by the *tat-2(qm179)* mutation can be partially rescued by transgenic expression of a cDNA coding for the mouse homologue ATP8B1 under the control of the endogenous *tat-2* promoter (P*tat-2::mAtp8b1*). Treating *clk-1(qm30); tat-2(qm179)* mutants that harbor the *Ptat-2:mAtp8b1* construct with RNAi against *mAtp8b1* abolished the rescue but had no effect by itself. The bars represent the mean defecation cycle of animals that have been scored for five consecutive defecation cycles. The error bars represent S.E.M. (n≥20 animals for each genotype). Differences were tested by a t-test; * represents P<0.05.

We previously carried out a genetic screen to find suppressors of the slow defecation phenotype of *clk-1* mutants [22]. In this screen we identified the *dsc-4/mtp* mutation (described in the Introduction) as well as another mutation, *dsc-3(qm179)*, which produced a very similar phenotype [22]. As the effects of *dsc-4/mtp* and *dsc-3(qm179)* are not additive (Table S1), they may act in a common pathway or affect a common process. We mapped *dsc-3(qm179)* between *dpy-13* and *unc-5* on LG IV [22]. Using the hypotheses that *dsc-3* is involved in lipoprotein metabolism (based on the identity of *dsc-4/mtp*) we identified *tat-2* as a candidate gene in that chromosomal region. We determined that *qm179* is allelic to *tat-2(tm1634)* based on the following experiments, whose results are shown in full in Table S1 (Table S1 lists all numerical values, samples sizes and statistical analyses for all defecation data shown in figures or mentioned in the text). Firstly, both RNAi against *tat-2* and the deletion mutation of *tat-2(tm1634)* were phenotypically similar to *qm179* in both the wild type and *clk-1* backgrounds. Secondly, the *tat-2(tm1634)* deletion mutation fails to complement *qm179* (Figure 1A). Thirdly, transgenic expression of *tat-2* rescues the suppression of *clk-1* by *qm179* (Figure 1A). Finally, a G-to-A point mutation that results in an amino acid change from Alanine to Threonine at residue 665 of the protein was found by sequencing the coding region of *tat-2* in *qm179* mutants (Figure 1B). We name the gene *tat-2* from this point on. The allele analyzed is always *qm179* except when otherwise specified.

### ATP8B1and TAT-2 are functional homologues

The high sequence conservation between TAT-2 and ATP8B1 suggests that their functions could be conserved as well. To test this directly we introduced a cDNA coding for mouse ATP8B1 in *clk-1;tat-2* mutants under the *C. elegans tat-2* promoter (Figure 1C). This could partially rescue the suppression of the defecation phenotype, and was abolished by RNAi against the mouse gene sequence (Figure 1C). Moreover, rescue by the mouse *Atp8b1* gene was also prevented by introduction of mutations corresponding to either the *tat-2(qm179)* mutation or the human G308V mutation (Table S1), strongly indicating a functional conservation.

### TAT-2 is required in the gut for its effect in *clk-1* mutants

In order to determine the focus of action of *tat-2*, we constructed a reporter gene in which the *tat-2* gene with 3.4 kb of upstream promoter sequence was fused in frame to *gfp*. This construct was capable of rescuing the defecation phenotype of *tat-2(qm179)* in the *clk-1* background (Table S1). The fusion protein was expressed in the gut, spermatheca, proximal gonad, vulva, excretory cell, excretory gland cell, pharyngeal procorpus, the pharyngeal-intestinal valve and the rectal gland cell (Figure S2), which is consistent with what was previously found by others [7], [8]. We also constructed three other reporters in which 3.4 kb of the *tat-2* promoter were replaced by the promoters from the intestinal specific *ges-1*, spermatheca-specific *sth-1*, or excretory canal-specific *pgp-12*, genes. Only the *Pges-1::tat-2::gfp* construct could rescue the phenotype (Table S1).

### The bile acid–binding resin cholestyramine suppresses the slow defecation of *clk-1* mutants

**Figure 2:**
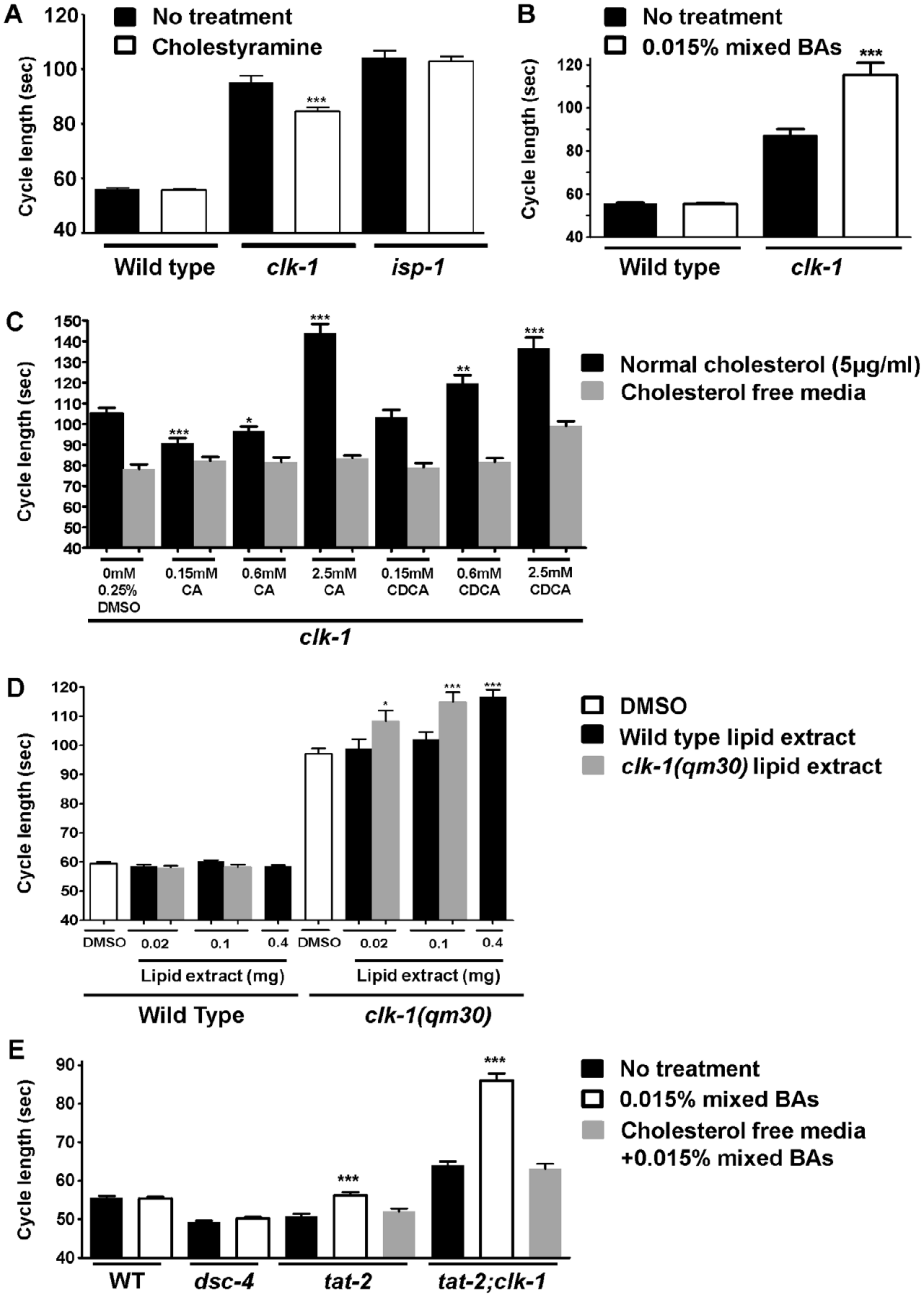
*clk-1* mutants are sensitive to the availability of endogenous BA–like molecules and to exogenous mammalian BAs. The bars represent the mean defecation cycle length of animals that had been scored for three consecutive cycles for *clk-1(qm30)* and *isp-1(qm150)* and for five consecutive defecation cycles for all other genotypes. (A) The BA-binding resin cholestyramine partially suppresses the slow defecation of *clk-1* mutants but is without effect on the wild type as well as on the mitochondrial mutant *isp-1(qm150)* (n≥50 for each genotype). (B) *clk-1(qm30)* mutants are sensitive to exogenous BAs, which enhance the *clk-1* phenotype (n≥20 animals). (C) Cholic acid (CA), a relatively hydrophilic BA, suppresses *clk-1* at very low concentration but enhances the phenotype at higher concentration. Chenodeoxycholic acid (CDCA) a relatively hydrophobic BA is without effect at 0.15 mM but enhances the phenotype at higher concentrations. All effects depend on the presence of cholesterol as they are totally or mostly (at 2.5 mM CDCA) abolished in the absence of cholesterol supplementation. The asterisks represent p values obtained by t-test comparing the defecation cycle after various treatments to that observed with 0.25% DMSO only. All treatments in the absence of cholesterol were indistinguishable from 0.25% DMSO except at 2.5 mM CDCA which was significantly slower at P<0.001; (n≥40 animals for each condition). (D) Ether extracts from the wild type or *clk-1* mutants enhance the defecation phenotype of *clk-1* mutants but not that of the wild type in a dose-dependent manner and extracts from *clk-1* mutants are approximately four times more active than extracts from the wild type. The asterisks represent t-test comparisons to the control of 0.25% DMSO treatment (n≥25 animals). (E) The defecation phenotype of *tat-2(qm179)* mutants can be rescued by exogenous BAs but this effect is abolished in the absence of cholesterol supplementation. In addition, the suppression of *clk-1* by *tat-2* is also suppressed by BA treatment, an effect which also requires cholesterol supplementation. BA treatment is without effect on the wild type and on *dsc-4* mutants. (n≥20 for each genotype and condition). The data presented in (B) and in (E) were generated simultaneously and the data for the wild type is the same in both panels. They are presented in separate panels for clarity. The error bars represent S.E.M. *** represents P<0.001, **represents P<0.01, *represents P<0.05.

Given the known function of ATP8B1 in bile acid secretion in mammals (see Introduction), and the fact that eliminating the function of *tat-2* suppresses *clk-1*, we wondered whether a pharmacological agent that targets BAs could also suppress *clk-1*. Cholestyramine is a BA-binding resin that is taken orally by people to lower the availability and thus the re-absorption of BA in the gut, which ultimately results in lowering in the level of circulating LDL [26]. We found that addition of 0.025% cholestyramine to worm plates partially suppresses the slow defecation cycle of *clk-1* mutants (Figure 2A). Cholestyramine had no effect on the wild type or on *isp-1* mutants, which, like *clk-1* mutants, have mitochondrial defects and a slow defecation cycle [27]. There was also no effect on *tat-2* or *dsc-4/mtp* mutants (Table S1). Cholestyramine can bind organic molecules of intermediate to low polarity that bear an acidic group. This supports the hypothesis that *C. elegans* secretes molecules that have chemical properties resembling those of BAs and that the altered defecation cycle of *clk-1* mutants is due to enhanced secretion of such molecules.

### Suppression by depletion of homologues of BA–biosynthetic enzymes

We reasoned that if there are mammalian-like BAs in worms they might be synthesized by enzymes that are similar to those in mammals. Reducing BA synthesis by depleting such enzymes by RNAi knock-down should suppress *clk-1*, similar to the effect of *tat-2* mutations and cholestyramine treatment. The biosynthesis of BAs in mammals is complex and involves a variety of enzymatic steps carried out in diverse cellular compartments [1]. In order to determine if BA-like molecules are synthesised in a similar manner in worms, we examined 17 of the most common of these steps by identifying the best *C. elegans* homologues of the mammalian enzymes, and testing their impact on the defecation cycle of *clk-1* mutants by RNAi (Table 1). Six of these enzymes are part of the same class of proteins, the P450 oxidases. As all the *C. elegans* proteins of this class are more or less equally similar to each of the vertebrate proteins, we tested all those we found by homology searching (78 genes). For other classes we tested several of the most homologous proteins (Table 1). Some classes of homologues did not have any effect on the defecation phenotype of *clk-1* mutants, e.g. 3β-hydroxy-Δ5-C27 steroid oxidoreductase, 2-methylacyl-CoA racemase, and bile acid CoA: amino acid N-acyltransferase. However, thirteen P450 enzymes as well as worm genes encoding proteins that are highly similar to mammalian branched-chain acyl-CoA oxidase and 3α-hydroxysteroid dehydrogenase, cholesterol 25-hydroxylase, bile acid CoA ligase, the D-bifunctional protein, and the two genes (*daf-22* and *nlt-1*) that separately encode the two activities of mammalian peroxisomal thiolase, were effective in affecting the defecation cycle of *clk-1* mutants (Table 1), suggesting that they may participate in the biosynthesis of BA-like molecules. Note that RNAi against *daf-9/cyp-22A1* and *hsd-1*, which encode activities that are known to participate in the synthesis of dafachronic acids, did not affect *clk-1* defecation (Table 1). *daf-12*, the nuclear receptor target of dafachronic acids, was also knocked down by RNAi under the same conditions as the enzymes: it produced only a very small, not significant, suppression (−2.8±3.9 seconds (P = 0.4795); n = 27 for the control, n = 38 for *daf-12(RNAi)*). Interestingly, in addition to suppressors of the phenotype, we also obtained a few enhancers, mostly among the P450s (Table 1). P450s in mammals have numerous functions besides BA synthesis, and thus have the potential to affect the rate of defecation in ways unrelated to the synthesis of BA-like molecules. This is consistent with the observation that most genetic changes that affect defecation tend to slow it down [21].

**Table 1:** Effects of RNA interference against *C. elegans* homologues of bile acid biosynthetic enzymes.

Enzyme Category#	*C. elegans* Genes	E-value from Blast Comparison to Mouse Protein	Summary of Effect on Defecation Rate	Effect on Defecation Rate (seconds)(p-values vs. control*)
Cholesterol 25-hydroxylase (AAC97482)	F35C8.5	3e-29	**↓**	−18.4±4.1 (P<0.0001)
	F49E12.10	3e-08	**-**	+8.4±5.5 (P = 0. 1)
	F49E12.9	2e-5	**-**	+7.8±5.3 (P = 0.15)
3β-Hydroxy-Δ5-C27 steroid oxidoreductase (AF277718_1)	*hsd-1*	1e-09	**-**	−1.8±4.0 (P = 0.67)
	*hsd-2*	4e-07	**-**	−4.9±5.3 (P = 0.41)
	*hsd-3*	2e-05	**-**	−4.7±4.8 (P = 0.33)
Bile acid CoA ligase (NP_036108)	*acs-20*	e-103	**↓**	−13.7±3.3 (P = 0.0001)
	*acs-22*	e-101	**-**	+2.4±4.2 (P = 0.57)
2-Methylacyl-CoA racemase (AAB72146)	C24A3.4	5e-59	**-**	−0.4±4.8 (P = 0.93)
	ZK892.4	3e-56	**-**	−2.5±4.2 (P = 0.56)
Branched-chain acyl-CoA oxidase (CAB65251)	*acox-1*	e-117	**-**	+1.1±4.4 (P = 0.79)
	F08A8.2	e-109	**-**	−0.2±4.1 (P = 0.97)
	F59F4.1	e-107	**↓**	−11.1±3.7 (P = 0.005)
	C48B4.1	e-105	**-**	+2.6±4.2 (P = 0.53)
	F08A8.4	e-104	**-**	−7.0±4.4 (P = 0.12)
	F08A8.3	e-101	**-**	−0.2±6.5 (P = 0.97)
D-bifunctional protein (CAA62015)	*dhs-28*	4e-84	**↓**	−18.8±4.7 (P = 0.0002)
	*dhs-25*	8e-15	**-**	+10.3±6.0 (P = 0.09)
	F54F3.4	1e-13	**-**	−2.1±6.4 (P = 0.75)
Peroxisomal thiolase 2 (AAA40098)	*daf-22*	e-134	**↓**	−18.7±3.6 (P<0.0001)
	*nlt-1*	2e-12	**↓**	−26.7±4.2 (P<0.0001)
B.A. CoA:a.a. N-acyltransferase (AAB58325)	W03D8.8	1e-27	**-**	−1.9±3.6 (P = 0.59)
	C31H5.6	2e-26	**-**	+1.6±4.2 (P = 0.70)
	K05B2.4	2e-25	**-**	−4.7±6.6 (P = 0.35)
	T05E7.1	1e-16	**-**	−2.6±4.7 (P = 0.58)
Δ4-3-Oxosteroid 5β-reductase (NP_663339) and 3α-Hydroxysteroid dehydrogenase (NP_085114)	Y39G8B.1	7e-72	**-**	+0.9±3.6 (P = 0.80)
	T08H10.1	3e-56	**↑**	+10.6±3.9 (P = 0.008)
	C07D8.6	2e-55	**↓**	−12.8±4.7 (P = 0.01)
	ZC443.1	2e-52	**-**	−8.7±4.3 (P = 0.06)
	Y39G8B.2	2e-47	**-**	−4.2±4.9 (P = 0.40)
	F53F1.3	5e-39	**-**	−5.2±5.6 (P = 0.36)
	F53F1.2	6e-38	**↓**	−15.4±6.3 (P = 0.02)
Cytochrome P450s	*cyp-13A1*	n/a	**-**	−8.9±5.5 (P = 0.12)
	*cyp-13A2*	n/a	**-**	−2.5±5.5 (P = 0.66)
	*cyp-13A3*	n/a	**-**	+10.5±6.3 (P = 0.11)
	*cyp-13A4*	n/a	**Not avail.**	
	*cyp-13A5*	n/a	**-**	−1.5±5.5 (P = 0.79)
	*cyp-13A6*	n/a	**-**	−2.8±5.9 (P = 0.64)
	*cyp-13A7*	n/a	**-**	−3.5±4.5 (P = 0.44)
	*cyp-13A8*	n/a	**-**	+2.0±6.8 (P = 0.77)
	*cyp-13A10*	n/a	**-**	+2.8±4.2 (P = 0.51)
	*cyp-13A11*	n/a	**-**	−7.6±5.5 (P = 0.18)
	*cyp-13A12*	n/a	**-**	−5.2±5.9 (P = 0.39)
	*cyp-13B1*	n/a	**-**	−7.4±5.8 (P = 0.21
	*cyp-13B2*	n/a	**-**	−4.3±6.6 (P = 0.52)
	*cyp-14A1*	n/a	**-**	+10.5±5.6 (P = 0.07)
	*cyp-14A2*	n/a	**-**	+6.3±6.6 (P = 0.34)
	*cyp-14A3*	n/a	**-**	−9.5±4.9 (P = 0.06)
	*cyp-14A4*	n/a	**-**	−4.9±5.7 (P = 0.40)
	*cyp-14A5*	n/a	**↓**	−14.0±4.2 (P = 0.002)
	*daf-9/cyp-22A1*	n/a	**-**	−0.5±3.3 (P = 0.87)
	*cyp-23A1*	n/a	**-**	+7.0±5.3 (P = 0.20)
	*cyp-25A1*	n/a	**-**	+2.4±4.4 (P = 0.58)
	*cyp-25A2*	n/a	**-**	+6.3±6.6 (P = 0.34)
	*cyp-25A3*	n/a	**-**	+0.3±5.2 (P = 0.96)
	*cyp-25A4*	n/a	**-**	+6.0±4.9 (P = 0.22)
	*cyp-25A5*	n/a	**-**	−2.0±4.3 (P = 0.64)
	*cyp-25A6*	n/a	**-**	−1.0±3.9 (P = 0.80)
	*cyp-29A1*	n/a	**-**	+10.0±6.8 (P = 0.16)
	*cyp-29A2*	n/a	**-**	−2.6±3.9 (P = 0.52)
	*cyp-29A3*	n/a	**↓**	−13.2±4.0 (P = 0.003)
	*cyp-29A4*	n/a	**-**	−0.2±3.9 (P = 0.96)
	*cyp-31A2*	n/a	**-**	+0.6±4.4 (P = 0.89)
	*cyp-31A3*	n/a	**↓**	−11.0±4.0 (P = 0.01)
	*cyp-32A1*	n/a	**-**	+1.4±4.6 (P = 0.75)
	*cyp-32B1*	n/a	**-**	+6.5±4.5 (P = 0.17)
	*cyp-33A1*	n/a	**-**	−3.6±4.7 (P = 0.45)
	*cyp-33B1*	n/a	**-**	+11.8±5.9 (P = 0.06)
	*cyp-33C1*	n/a	**-**	−6.2±4.3 (P = 0.16)
	*cyp-33C2*	n/a	**-**	−4.6±4.1 (P = 0.27)
	*cyp-33C3*	n/a	**-**	−5.9±4.9 (P = 0.24)
	*cyp-33C4*	n/a	**-**	−5.0±3.9 (P = 0.20)
	*cyp-33C5*	n/a	**↓**	−8.0±3.4 (P = 0.024)
	*cyp-33C6*	n/a	**↓**	−13.1±3.4 (P = 0.0005)
	*cyp-33C7*	n/a	**-**	+3.4±4.6 (P = 0.46)
	*cyp-33C8*	n/a	**↑**	+12.1±4.5 (P = 0.01)
	*cyp-33C9*	n/a	**-**	+7.3±5.1 (P = 0.17)
	*cyp-33C11*	n/a	**-**	+1.6±4.6 (P = 0.74)
	*cyp-33C12*	n/a	**-**	+9.8±5.2 (P = 0.07)
	*cyp-33D1*	n/a	**↓**	−8.9±3.5 (P = 0.02)
	*cyp-33D3*	n/a	**-**	−4.8±4.6 (P = 0.31)
	*cyp-33E1*	n/a	**↑**	+12.6±5.4 (P = 0.03)
	*cyp-33E2*	n/a	**-**	−2.2±5.3 (P = 0.69)
	*cyp-33E3*	n/a	**-**	+4.0±6.7 (P = 0.55)
	*cyp-34A1*	n/a	**-**	+1.2±6.2 (P = 0.84)
	*cyp-34A2*	n/a	**-**	+0.5±5.7 (P = 0.93)
	*cyp-34A3*	n/a	**-**	+1.0±4.0 (P = 0.80)
	*cyp-34A4*	n/a	**↓**	−14.5±4.7 (P = 0.003)
	*cyp-34A5*	n/a	**-**	+2.4±4.6 (P = 0.61)
	*cyp-34A6*	n/a	**↓**	−11.5±5.0 (P = 0.03)
	*cyp-34A7*	n/a	**-**	+1.1±4.2 (P = 0.80)
	*cyp-34A8*	n/a	**-**	+5.6±4.4 (P = 0.22)
	*cyp-34A9*	n/a	**↓**	−12.5±5.2 (P = 0.02)
	*cyp-34A10*	n/a	**-**	+3.7±4.5 (P = 0.41)
	*cyp-35A1*	n/a	**-**	+4.6±6.3 (P = 0.48)
	*cyp-35A2*	n/a	**-**	−2.9±5.8 (P = 0.62)
	*cyp-35A3*	n/a	**-**	−3.9±5.2 (P = 0.46)
	*cyp-35A4*	n/a	**↓**	−12.9±4.1 (P = 0.004)
	*cyp-35A5*	n/a	**-**	+5.0±6.8 (P = 0.47)
	*cyp-35B1*	n/a	**-**	+9.3±6.9 (P = 0.19)
	*cyp-35B2*	n/a	**-**	+1.7±6.6 (P = 0.80)
	*cyp-35B3*	n/a	**-**	+12.1±7.0 (P = 0.1)
	*cyp-35C1*	n/a	**-**	+10.7±6.6 (P = 0.12)
	*cyp-35D1*	n/a	**-**	−1.3±6.6 (P = 0.84)
	*cyp-36A1*	n/a	**-**	+3.5±5.3 (P = 0.51)
	*cyp-37A1*	n/a	**↑**	+14.4±5.4 (P = 0.01)
	*cyp-37B1*	n/a	**-**	+8.7±4.4 (P = 0.06)
	*cyp-42A1*	n/a	**-**	−2.8±4.4 (P = 0.52)
	*cyp-43A1*	n/a	**-**	+4.7±7.0 (P = 0.51)
	*cyp-44A1*	n/a	**-**	+0.6±3.5 (P = 0.86)

To identify *C. elegans* genes involved in BA synthesis, we carried out Blast homology searches for the translated products of mouse genes known to be involved in the process. In one case, the Blast results of two mouse proteins (NP_663339 and NP_085114) identified the same in worm homologues. The most homologous genes (shown in the “*C. elegans* genes” column) were tested by treatment of *clk-1(qm30)* mutants with RNAi against these genes. All cytochrome P450s were screened as they are highly similar to each other. The defecation rates are given as means ± S.E.M.

# The accession numbers of the proteins used for the search are given.

*The p-values were obtained by comparing to control *clk-1* mutants grown in parallel on HT115 bacteria harbouring only the empty pPD129.36 vector.

### Suppression of *clk-1* by *daf-36* suggests that the molecules affected by TAT-2 and cholestyramine are cholesterol derivatives

DAF-36 is a Rieske oxygenase that acts as a cholesterol 7-desaturase that converts cholesterol to 7-dehydrocholesterol [28], [29]. DAF-36 is necessary for dafachronic acid biosynthesis, which is why mutation of *daf-36* leads to a dauer constitutive phenotype. We found that *daf-36(k114)* also suppresses the slow defecation cycle of *clk-1* (by 19.4 seconds), with only a very small effect (1.5 seconds) on the wild type (Table S1). This is consistent with the hypothesis that the active molecules that are transported by TAT-2 and are bound by cholestyramine could be oxidized cholesterol derivatives, although they are clearly distinct from dafachronic acids (see above).

### *clk-1* mutants but not wild-type animals are sensitive to exogenous BAs

Lowering the level of the hypothetical BA-like molecules by reducing their secretion via mutation of *tat-2*, reducing their biosynthesis by RNAi or mutations against potential biosynthetic enzymes, or by sequestration through cholestyramine suppresses the *clk-1* phenotype. We reasoned that the phenotype might therefore be enhanced by BA supplementation. We treated *clk-1* mutants with mixed mammalian BAs and found that their phenotype was indeed enhanced while the wild type was completely insensitive (Figure 2B). These findings show that externally applied BAs can act on *C. elegans*. It also suggests that in the wild type the processes that are affected by BAs and that ultimately determine the defecation cycle, such as cholesterol handling (see below) and lipoprotein metabolism (see Introduction), are better regulated than in *clk-1* mutants.

### The effects of exogenous BAs depend on their structures

In mammals, BAs of different structures have been found to interact differently with nuclear hormone receptors thus affecting differently the regulation of BA synthesis and secretion, and also to be more or less efficient in cholesterol uptake [2]. In particular, more hydrophobic BAs appear to result in greater cholesterol uptake [30]. To test whether the structures of the BAs are important for their effects on *clk-1* mutants we treated the wild type and *clk-1* mutants with three concentrations of cholic acid (CA), one of the main relatively hydrophilic mammalian BA, and chenodeoxycholic acid (CDCA), one of the main relatively hydrophobic mammalian BA. No treatment had any effect on the wild type (Table S1). However, at two concentrations (0.15 mM and 0.6 mM), CA suppressed the defecation cycle of *clk-1* mutants, although it enhanced the phenotype at 2.5 mM, while CDCA enhanced the phenotype in a dose-dependent manner at all concentrations tested (Figure 2C). One possibility to explain the ability of CA to suppress *clk-1* suggests that it might be more hydrophilic than the average BA-like molecules secreted by worms, thus effectively diluting their strength in taking up cholesterol. This notion is also supported by the observation that CA was a better suppressor at lower (0.15 mM) than at the higher (0.6 mM) concentration, and enhanced the phenotype at the highest concentration (2.5 mM). This suggests that at the higher concentrations the greater amount of BA (here CA) provided by the treatment in part compensates for the fact that CA is a more hydrophilic BA. CDCA had no effect at the lowest concentration but enhanced the phenotype at higher concentrations (Figure 2C).

### An activity that alters the defecation cycle of *clk-1* mutants but not that of the wild type can be extracted from *C. elegans* and is more abundant in *clk-1* mutants

The results presented above suggest that *C. elegans* produces and secretes molecules with BA-like properties, and possibly structures, and that this process is deregulated in *clk-1* mutants. We reasoned that the hypothetical endogenous BA-like molecules should have the same effect on the wild type and *clk-1* mutants as exogenous BAs. To test this we made lipid extracts [31] from both the wild type and *clk-1* mutants and assayed them on the defecation cycle of both genotypes. The lipid extracts were applied to plates in the same way as BAs in previous experiments. Extracts from both genotypes had no effect on the defecation of the wild type. However, extract from *clk-1* mutants at 0.02 and 0.1 mg significantly enhanced the phenotype of *clk-1* mutants (Figure 2D). At these concentrations wild type extracts had no significant effect on the mutants. Thus to establish that the wild type also contains the activity, and to measure how much higher the activity was in *clk-1* mutants, we produced a large quantity of extract from the wild type, which allowed to test 0.4 mg of activity on the wild type and *clk-1*. The high concentration of wild type extract was again ineffective on wild type animals but enhanced the phenotype of *clk-1* as much as 0.1 mg of extract from *clk-1* (Figure 2D). We conclude that both the wild type and *clk-1* mutants contain the activity but that *clk-1* mutants contain approximately 4× time higher steady-state levels of the activity.

### *clk-1* and *clk-1;tat-2* mutants show altered cholesterol content

**Figure 3:**
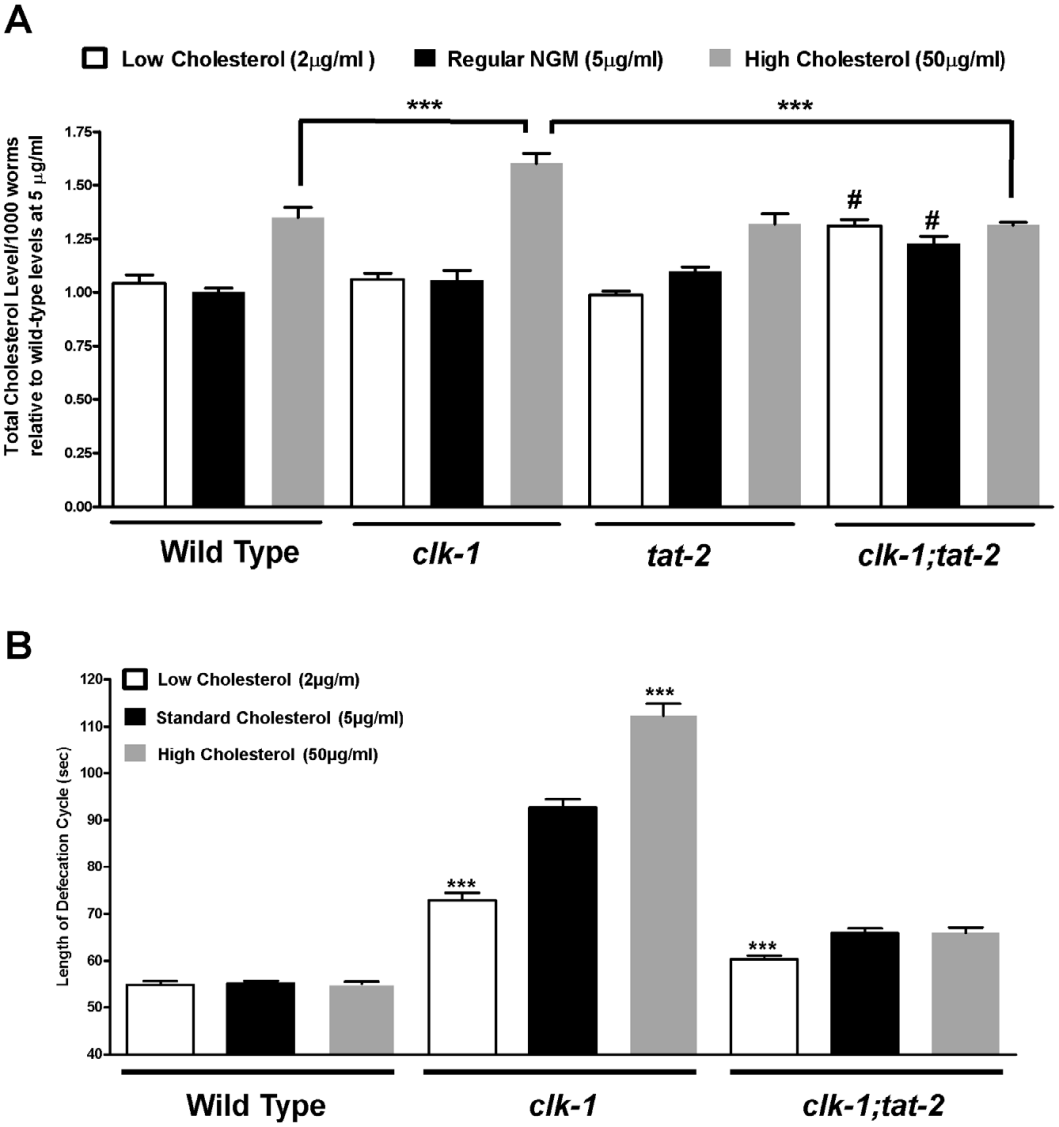
The effect of different levels of cholesterol supplementation on mutant genotypes. (A) Total cholesterol content of worms was measured in the wild type and in *clk-1* mutants previously grown under different regimes of cholesterol supplementation. High level of cholesterol supplementation induced greater cholesterol content than lower levels in all genotypes except *clk-1; tat-2*. High level of cholesterol supplementation produced the highest cholesterol content in *clk-1* mutants, an effect that was fully suppressed by *tat-2* in *clk-1; tat-2* mutants. In general the cholesterol content of *clk-1; tat-2* was high and independent of cholesterol supplementation. Error bars represent the standard errors of the means across repeated experiments (n≥16). Each sample represented 1000 worms and was measured three times. Cholesterol levels are shown as normalized to the wild type level under 5 µg/ml supplementation. The wild type level was 0.056 µM. *** represents P≤0.001 for t-test comparison as indicated, #represents P<0.001 for t-test comparisons to the wild type cholesterol content at the same level of cholesterol supplementation. Table S2 lists all numerical values and further statistical tests. (B) The slow defecation phenotype of *clk-1(qm30)* mutants is sensitive to both low and high cholesterol supplementation. The phenotype is suppressed by low and enhanced by high supplementation. The sensitivity to high supplementation is suppressed by *tat-2(qm179)* in *clk-1; tat-2* double mutants. *tat-2(qm179)* attenuates the effects that different levels of supplementation have on *clk-1*. The bars represent the means of the defecation cycle lengths of n≥25 animals, each scored for three consecutive defecation cycles for the *clk-1(qm30)* mutants and for five consecutive defecation cycles for all other genotypes. The error bars represent the standard error of the mean. The asterisks indicate for each genotype whether the defecation cycle length with low or high cholesterol supplementation is significantly different from that with the standard level of cholesterol by a t-test. *** represents P≤0.0001.

One of the functions of BAs is to regulate cholesterol uptake and handling. We therefore measured the level of cholesterol in the wild type and in *clk-1* mutants grown under low (2 µg/ml), standard (5 µg/ml) and high (50 µg/ml) levels of cholesterol supplementation. Both the wild type and *clk-1* mutants grown on high cholesterol contained significantly more cholesterol than when grown under standard conditions (Figure 3A). However the increase was significantly greater in *clk-1* mutants. There was no significant difference between 2 µg and 5 µg/ml of supplementation for either genotype. We also assayed the cholesterol content of *tat-2* and *clk-1;tat-2* mutants. The cholesterol content of *tat-2* was similar to that of the wild type at all levels of cholesterol supplementation. However the increase of cholesterol content observed in *clk-1* mutants under high cholesterol supplementation was fully abolished in *clk-1;tat-2* double mutants (Figure 3A and Table S2). Furthermore, cholesterol content in the double mutants was elevated at low and standard level of supplementation and thus similar at all levels of cholesterol supplementation, indicating that *clk-1* and *tat-2* interact in determining the level of cholesterol uptake and content.

### The *clk-1* phenotype is sensitive to high cholesterol supplementation in a *tat-2*–dependent manner

We have previously shown that the defecation cycle of *clk-1* mutants, but not that of the wild type, is suppressed by lowering the levels of dietary cholesterol from 5 µg/ml to 2 µg/ml [23]. We have now extended this observation to the effect of high cholesterol (50 µg/ml), which has no effect on the wild type but further slows down the defecation of *clk-1(qm30)* mutants (Figure 3B). We had observed (Figure 3A) that *clk-1* and *tat-2* interact in determining the level of cholesterol uptake. We therefore wondered if the metabolism of the BA-like molecules was involved in these effects of the level of dietary cholesterol on the defecation cycle. We found that low cholesterol shortened the defecation cycle of *clk-1;tat-2*, but that the effect of high cholesterol on *clk-1* mutants was fully suppressed in *clk-1;tat-2* mutants (Figure 3B). The observation that altering the level of media cholesterol can affect the defecation phenotype of *clk-1* mutants in both directions suggests that uptake or subsequent handling of cholesterol can change the phenotype caused by the deregulated metabolism of the BA-like molecules in *clk-1* mutants.

### Exogenous BAs rescue *tat-2* and enhance *clk-1* in a cholesterol-dependent manner

The results described above suggest that the suppression produced by the *tat-2* mutation might be due to lower secretion of the BA-like molecules. To test this directly we treated *tat-2* and *clk-1;tat-2* mutants with a small amount (0.015%) of mixed mammalian BAs (Figure 2E and Table S1). These exogenous BAs had no effect on the wild type or *dsc-4* mutants but rescued the *tat-2* phenotype in both the wild-type and *clk-1* backgrounds. Furthermore, these effects of the exogenous BAs were abolished in the absence of cholesterol supplementation (Figure 2E). We also found that the effects of BAs we have previously observed, such as suppression and enhancement of *clk-1* by pure CA or CDCA at various concentrations require cholesterol supplementation (Figure 2C). These results indicate: 1) that the effect of *tat-2* on *clk-1* mutants is mediated by a reduction in the secretion of BA-like molecules; and 2) that the effects of BAs and *tat-2* on *clk-1* mutants implicate changes in cholesterol uptake.

### Mitochondrial oxidative stress is responsible for the slow cycle of *clk-1* mutants

We have shown above that the phenotypes of *clk-1* mutants include deregulated metabolism of BA-like molecules, which results in altered cholesterol content and abnormal sensitivity to the level of cholesterol supplementation. Previous studies of *clk-1* indicated that the principal cellular defect of these mutants is an elevated level of mitochondrial oxidative stress, characterized by elevated mitochondrial ROS production [18], elevated oxidative damage [32], [33], and increased sensitivity to pro-oxidant drugs [34]. In addition, several of the *clk-1* phenotypes are strongly enhanced when the expression of the main mitochondrial superoxide dismutase (SOD-2) is reduced by RNAi [32] or mutation [33]. In fact defecation was among the phenotypes that were found to be enhanced in the *clk-1*;*sod-2* double mutants [33].

**Figure 4:**
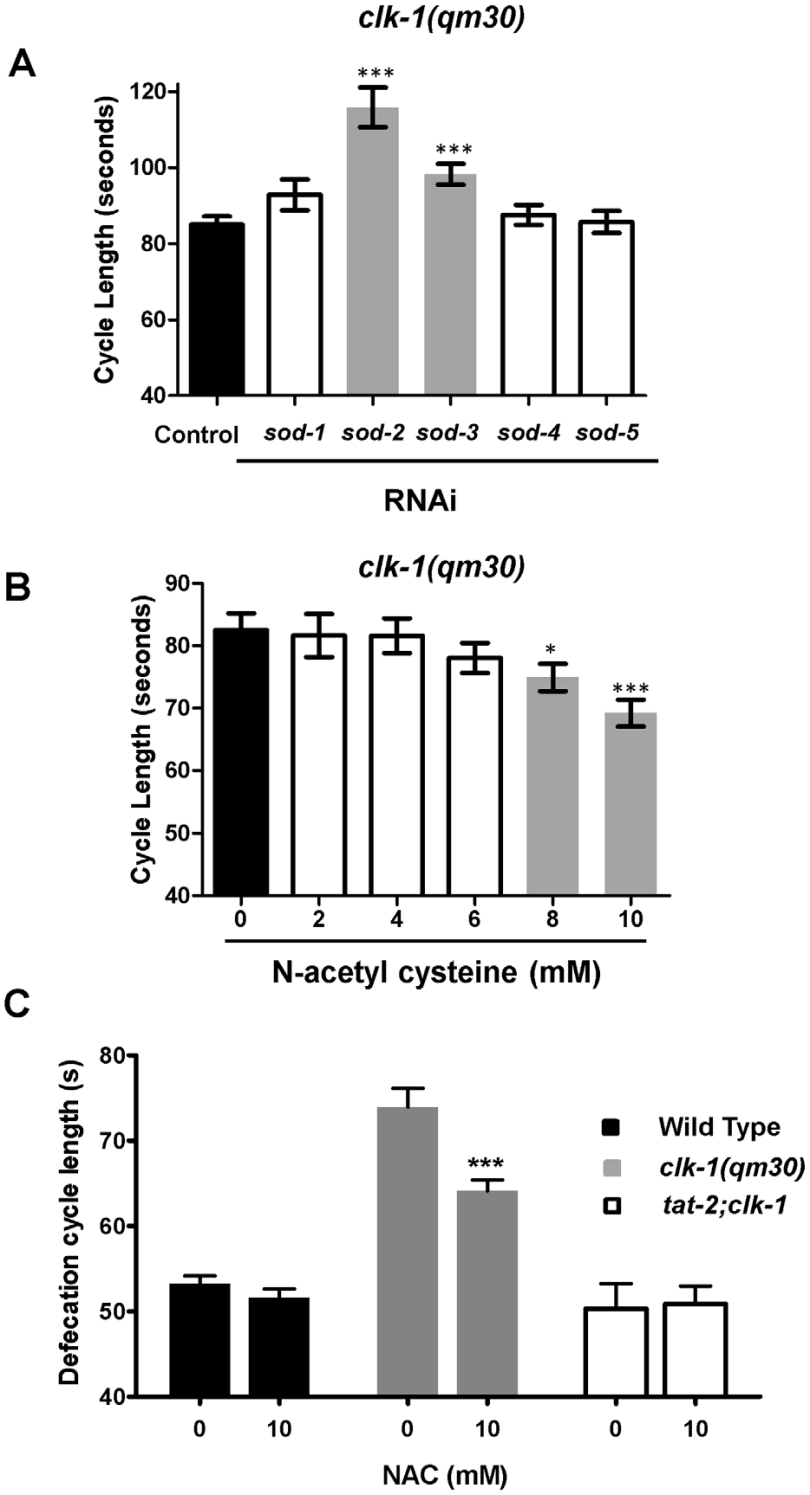
*clk-1* mutants are sensitive to alterations in ROS metabolism. The bars represent the mean defecation cycle length of animals that were scored for one cycle. (A) RNAi mediated knock-down of the mitochondrial localized superoxide dismutases SOD-2 and SOD-3 enhance the slow defecation cycle phenotype of *clk-1* mutants (n≥6 for each genotype). (B) NAC (N-acetyl cysteine) supplementation suppresses the slow defecation of *clk-1* mutants at 8 and 10 mM concentrations (n≥22 for each genotype). (C) Treatment with 10 mM NAC is without effect on *tat-2; clk-1* double mutants (n≥6 for each genotype). The error bars represent S.E.M. *** represents P<0.001, **represents P<0.01, *represents P<0.05.

To further explore the link between ROS and the *clk-1* defecation phenotype we first determined whether RNAi against the other *C. elegans sod* genes had any effect. We found that in addition to *sod-2*, RNAi knockdown of *sod-3*, the gene encoding the other mitochondrial superoxide dismutase, enhanced the defecation phenotype of *clk-1* (Figure 4A). However, RNAi against the three non-mitochondrial *sod* genes (*sod-1*, *sod-4*, and *sod-5*) did not affect the phenotype (Figure 4A), indicating that the enhancement of the phenotype is specific to alterations in mitochondrial ROS levels. Consistent with previous findings, this suggests that the slow defecation phenotype of *clk-1* mutants might be due to their elevated mitochondrial ROS production. In order to test this further we treated *clk-1* mutants with the antioxidant N-acetyl-cysteine (NAC) a commonly used hydrophilic antioxidant, which can reduce mitochondrial ROS production [18]. We found that NAC treatment could partially suppress the slow defecation cycle in a dose-dependent manner (Figure 4B). Complete suppression could not be obtained because higher levels of the compound was toxic, possibly because of inhibition of normal ROS levels in other compartments. Finally, to test whether the increased mitochondrial oxidative stress is the cause of the deregulated metabolism of the BA-like molecules we tested whether the *tat-2(qm179)* mutation could suppress the effect of antioxidant treatment. We found that treatment with 10 mM NAC was without effect on *tat-2; clk-1* (Figure 4C and Table S1), indicating that *tat-2(qm179)* is epistatic to antioxidant treatment. This is consistent with the elevated mitochondrial oxidative stress being the primary cause of the deregulation of the metabolism of the BA-like molecules observed in *clk-1* mutants.

### Mitochondrial oxidative stress is responsible for the increased level of activity in lipid extracts from *clk-1* mutants

**Figure 5:**
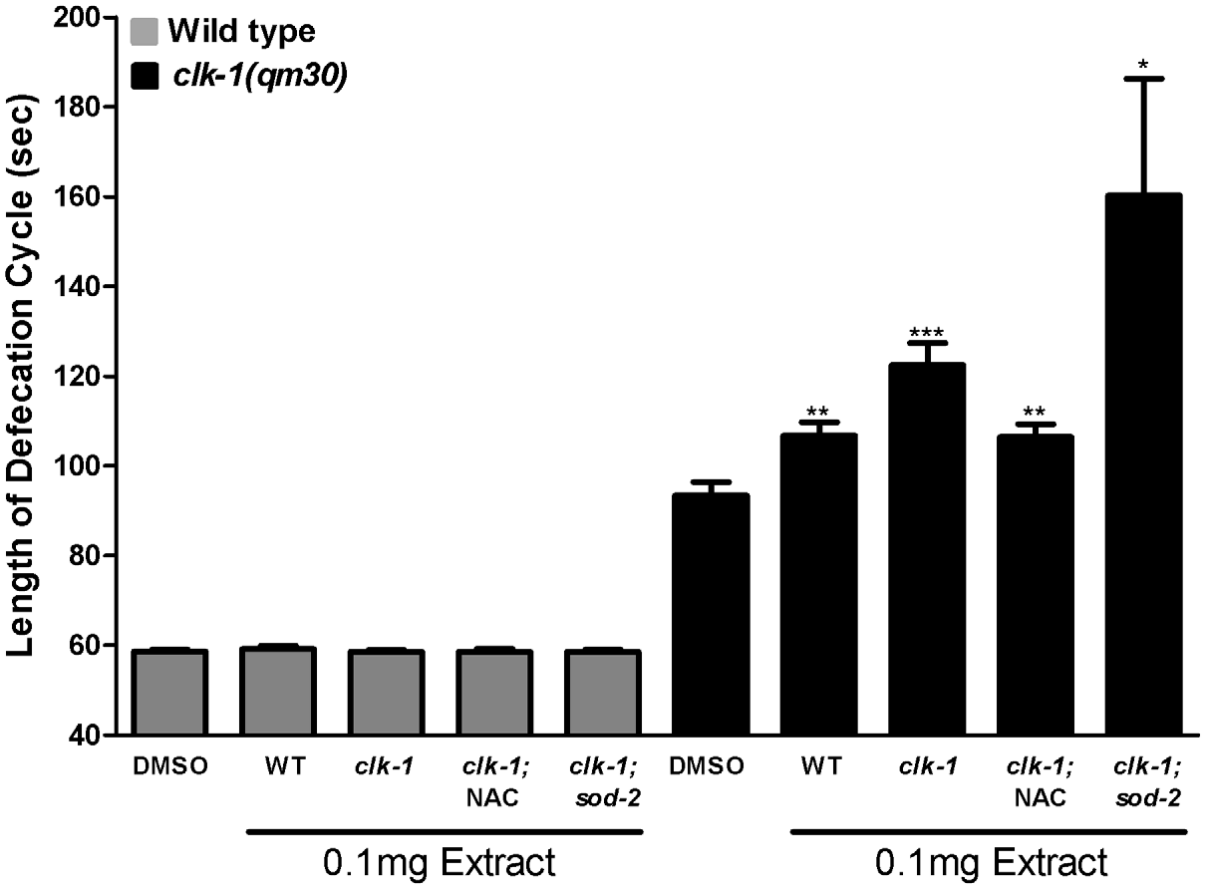
The activities of endogenous BA–like molecules are altered by mitochondrial ROS levels. The bars represent the mean defecation cycle lengths. As before (Figure 2D), we find that *clk-1* mutant lipid extracts contain more activity than the wild type. However, treatment with NAC lowers the activity level to that of the wild type extract, and the extract from *clk-1; sod-2* double mutants has much more activity than the extract from *clk-1* single mutants. All extracts are without effect on the wild type. The asterisks represent t-test comparisons to the control of 0.25% DMSO treatment (n≥25 animals). The error bars represent S.E.M. *** represents P<0.001, **represents P<0.01, *represents P<0.05.

The hypothesis suggested by the results described so far is that the *clk-1* defecation phenotype is the result of increased mitochondrial oxidative stress in these mutants, which increases the level of activity of BA-like molecules. We tested this hypothesis directly by producing and testing lipid extracts from *clk-1* mutants treated with NAC and from *clk-1(qm30);sod-2(ok1030)* double mutants (Figure 5). NAC treatment reduced the level of the activity found in the extract, and the extract from *clk-1;sod-2* double mutants contained substantially higher level of activity than the *clk-1* extract. For an unknown reason the *clk-1;sod-2* extract was the most variable in terms of its activity on individual worms (Figure 5 and Table S1).

## Discussion

Here we have shown that: 1) *clk-1* mutants are suppressed by mutations of TAT-2, the worm orthologue of an ATPase that is necessary for BA secretion in mammals, 2) the suppression by *tat-2* can be rescued by exogenous BAs, 3) RNAi knockdown of several *C. elegans* enzymes homologous to those that are implicated in BA synthesis in mammals suppress the *clk-1* phenotype, but not the knockdown of some of the enzymes known to be necessary for dafachronic acid synthesis, 4) *clk-1* mutants display a cholesterol-dependent sensitivity to exogenous BAs, as well as a sensitivity to cholestyramine, a drug that sequesters BAs, 5) *clk-1* mutants but not the wild type are sensitive to an activity contained in lipid extracts from worms, 6) the *clk-1* defecation phenotype is suppressed by a mutation in *daf-36*, which encodes a cholesterol 7-desaturase, suggesting that the activity is a cholesterol derivative, 7) *clk-1* mutants contain more of this activity, 8) the level of the activity is altered by mitochondrial oxidative stress, 9) *clk-1* mutants have a deregulated cholesterol metabolism, as indicated by the fact that their phenotype can be affected by reducing or increasing the level of dietary cholesterol and that they accumulate more cholesterol than the wild type when supplied with high levels of dietary cholesterol, 10) *clk-1* and *tat-2* interact in determining cholesterol content as, in contrast to what is observed in the wild type, the cholesterol content of *clk-1;tat-2* is similar at all levels of dietary cholesterol supplementation. This last observation suggests that the abnormal cholesterol metabolism is caused by the deregulated metabolism of the BA-like molecules that are affected by *clk-1* and *tat-2*. Together all these observations imply that there are BA-like molecules involved in cholesterol uptake in *C. elegans*, but also that these molecules are likely to be structurally similar to BAs, as their biosynthesis and secretion are affected by activities that are known to affect BAs in mammals.

**Figure 6:**
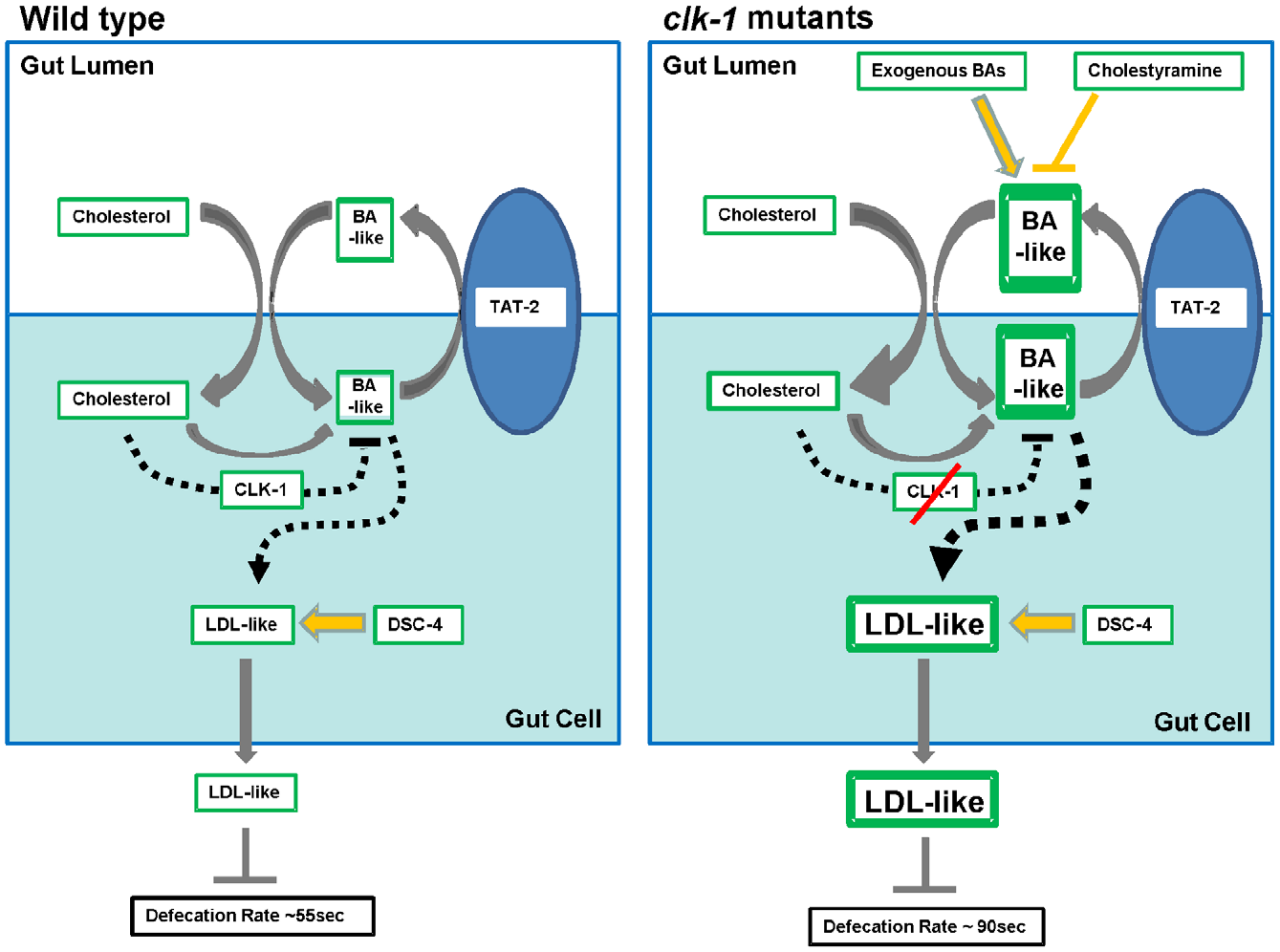
Schematic representation of the biosynthetic and regulatory interactions relating cholesterol uptake and bile acid (BA) biosynthesis and secretion to the biosynthesis and secretion of LDL-like lipoproteins. See Discussion for further explanation.

The results summarized in the previous paragraph lead to a model of regulatory relationships between cholesterol availability, cholesterol uptake, the synthesis and secretion of BA-like molecules, and LDL-like lipoprotein synthesis and secretion in *C. elegans* (Figure 6). All our findings appear to be remarkably consistent with what is known about the synthesis and regulation of BAs and LDL in vertebrates. Thus we propose that secreted BA-like molecules participate in cholesterol uptake and that the function of TAT-2 is required for their secretion. Cholesterol is used in the synthesis of the BA-like molecules and, as in mammals, the BA-like molecules act directly on cholesterol uptake but also as signalling molecules that positively regulate the synthesis of LDL-like lipoproteins. The core of our model is that CLK-1, via its effect on limiting mitochondrial ROS generation, is required for a negative feedback mechanism that down-regulates the synthesis of the BA-like molecules as a function of cholesterol uptake. In the absence of CLK-1 more BA-like molecules are synthesized (Figure 2D) and more cholesterol can be taken up (Figure 3A). The increased synthesis of the BA-like molecules up-regulates the level of LDL-like lipoprotein synthesis and secretion, which in turn determines the length of the defecation cycle.

Our data show that availability of BA-like molecules and the rate of defecation are tightly linked as shown by the sensitivity of the mutant defecation cycle to BA supplementation, sequestration of the BA-like molecules, and the inhibition of the synthesis of the BA-like molecules. The hypothesis that CLK-1 is necessary for a feed-back from cholesterol uptake to the synthesis of the BA-like molecules provides the link between the level of cholesterol supplementation and the level of the BA-like molecules (and thus between the level of cholesterol supplementation and defecation) (Figure 6). However, the model cannot accurately predict the effect of mutations on the level of whole-animal cholesterol. Indeed, the level of cholesterol likely depends on cholesterol flux through the entire organism. This is determined by a number of factors that we cannot precisely quantify at this stage, including the exact quantitative relationship between the level of cholesterol uptake and the level of synthesis of the BA-like molecules via the CLK-1-dependent mechanism, the level of cholesterol loss through the synthesis of BA-like molecules if these are cholesterol-derived, and the loss of the BA-like molecules through secretion, the level of cholesterol loss through LDL-like lipoprotein secretion (whose target in the organism is unknown), the level of cholesterol loss through yolk synthesis and egg-laying, and in fact any other form of cholesterol elimination or storage, whether or not regulated by the BA-like molecules.

Suppression of the *clk-1* defecation phenotype can be obtained by knocking down the enzymes necessary for peroxisomal β-oxidation that in mammals are necessary for shortening the side-chain of cholesterol (Table 1). This suggests that if the *C. elegans* BA-like molecules are cholesterol derived they might have a shortened side-chain. This is in contrast to dafachronic acid (Figure S1), which is a steroid that acts as a hormone that regulates development in *C. elegans* [35]. We have not yet tested if the BA-like molecules can affect other *clk-1* phenotypes in addition to defecation, such as slow aging. More detailed structural information on the *C. elegans* BA-like molecules, and possibly the availability of synthetic molecules, might be necessary to test rigorously their effect on phenotypes that are harder to quantify than defecation.

The suppressive effect of cholic acid (CA) at very low concentrations is difficult to explain unless the BA-like molecules are indeed structurally similar to BAs. However, if this is the case the observed effect might result from the dilution by CA of the native and potentially more hydrophobic BA secreted by worms. However, as CA serves as negative feedback for BA synthesis and secretion in mice [36], it is possibly that it could carry out a similar role in *C. elegans*, which would provide an alternative explanation for its paradoxical action at low concentration. If this is the case, further study of this phenomenon might help in identifying the nuclear hormone receptors (NHRs) through which the *C. elegans* BAs might regulate metabolism and their own synthesis. We have already identified a number of nuclear hormone receptor loci whose down-regulation suppresses *clk-1* mutants (not shown). One or several of these could be the receptors for the BA-like molecules.

The metabolic syndrome is a collection of age-associated disease risk factors that includes obesity, insulin resistance, hypertension and dyslipidemia. Oxidative stress, which is well known to increase with age and in obese individuals [37], has been implicated in most of the components of the metabolic syndrome and might be the common link between them [38], [39], [40]. Our findings with *C. elegans*, where there appears to be BA-like molecules whose synthesis, secretion and activity shares strong similarities with BAs in mammals, suggest that mitochondrial oxidative stress can lead to deregulation of BA synthesis. Abnormal BA levels in turn could lead to metabolic disease processes via the action of BAs on sterol, lipid and glucose metabolism by signalling through BA receptors. Interestingly, the possibility of an involvement of oxidative stress on the regulation of BA synthesis and thus on the consequences of a deregulation of this process has not yet been explored in mammals.

## Materials and Methods

### General methods

Fourth larval stage (L4) animals were transferred to the test plates and grown at 20°C. The effects of the different cholesterol concentrations or compounds were scored after raising the worms on the test plates for one generation. Defecation cycle rates were measured as previously described [22], at 20°C for all experiments except for the RNAi and antioxidant treatments for which 25°C was used. Compounds (cholestyramine, mixed bile acids, cholic acid, and chenodeoxycholic acid were tested by spreading them on plates, except that N-acetyl-L-cysteine was added to the nematode growth media (NGM) prior to pouring it into plates. See also Text S1.

### Positional cloning of *dsc-3(qm179)*

*dsc-3* had previously been mapped to LG IV, between *unc-33* and *dpy-4* [22]. By using 2-point and 3-point mapping strategies, the genetic position of *qm179* was refined to a position between the two cloned gene *dpy-13* and *unc-5*. Due to the incomplete cosmid coverage of the *tat-2* gene, no cosmid that spans this region can rescue the *qm179* mutants. Therefore *qm179* mutants were rescued by injecting two partially overlapping PCR fragments of *tat-2* genomic DNA (from −3123 to +7277 and from +7252 to +13567, which includes the UTRs) for in vivo recombination. Two other mutations allelic to *qm179* had been originally identified, *qm180* and *qm184* [22]. The lesion in *qm184* was identical to the *qm179* lesion, and the lesion in *qm180* was not found in the *tat-2* exonic sequences. The *tat-2(tm1634)* allele was obtained from the National Bioresource Project and outcrossed three times. See also Text S1.

### Construction of plasmids and transgenic strains

The *tat-2* transcriptional reporter, *Ptat-2::gfp* (pCDB898) was used as backbone to build the *Ptat-2::mAtp8b1*, *Ptat-2::mAtp8b1 A705T*, *Ptat-2::mAtp8b1 G308V* clones. The PCR product of 3.4 kb upstream of the initiating ATG of *tat-2* was cloned into the PstI and SmaI sites of the pPD95_77 vector. The full length of mouse *Atp8b1* cDNA was amplified from the RIKEN clone F830210O18.

To construct *Ptat-2::tat-2::gfp* (pCDB902) a 3945 bp long wild type *tat-2* cDNA containing 22 exons was inserted into the SmaI site of pCDB898. To construct *Pges-1::tat-2::gfp*, *Psth-1::tat-2::gfp*, and *Ppgp-12::tat-2::gfp*, (pCDB906, pCDB905 and pCDB904, respectively) the *tat-2* promoter of pCDB902 was replaced by PCR products of 2 kb upstream of the *ges-1* initiation codon, 1.6 kb upstream of the *sth-1* initiation codon or 2.7 kb upstream of the *pgp-12* initiation codon. These constructs were injected into *clk-1; tat-2(qm179)* mutants at a concentration of 0.1 ng/µl along with the transformation marker *ttx-3::gfp* at a concentration of 200 ng/µl. See also Text S1.

### Total cholesterol content

Lipids were extracted following [41], and the cholesterol content was determined with a kit (10007640) from Cayman Chemical. The final concentration of Triton X-100 in each sample was 0.5%. We also measured the volumes of young adults for all genotypes as previously described [42], and no difference from the wild type was found (data not shown). See also Text S1.

### Active lipid extracts

The lipid extracts were prepared as previously described [31] and re-suspended in DMSO. To assay the activity of extracts from the wild type, *clk-1(qm30)*, *clk-1(qm30); sod-2(ok1030)* or *clk-1* mutants treated with NAC, 36 µl of DMSO-dissolved extract (or 36 µl of DMSO as control) was spread onto 5 cm plates. Phenotypes of adult progeny were measured after raising L4 animals on the test plates for one generation. Due to the sensitivity of *clk-1* mutants to dietary cholesterol level, we measured and calculated that the final concentrations of extracts applied to the plates contained less than 0.1 µg/ml of cholesterol, which cannot therefore be responsible for any of the effects observed (Figure 2D). See also Text S1.

### RNAi feeding

5–10 *clk-1(qm30)* hermaphrodites L4 larvae were picked to RNAi plates. For the following 3 days, worms were transferred to new RNAi plates to rid of contaminating OP50 bacteria. Progeny worms were grown to the L4 stage and were then picked to new RNAi plates for scoring. 18 hours later, they were transferred to 25°C. After two hours of acclimation, their defecation phenotype was scored. We used 25°C for all RNAi experiments, except those shown in Figure 1C, because the responses tend to be more robust [22]. For each RNAi clone, five worms were scored for one defecation cycle. Clones that had a significant effect on defecation rate were re-screened 2–3 times.

## Supporting Information

Figure S1 The structures of (A) cholesterol, (B) chenodeoxycholic acid (CDCA), (C) cholic acid (CA), and (D) dafachronic acid.
(PDF)

Figure S2 The expression pattern of the translational fusion reporter *tat-2::gfp*. The expression of TAT-2 was first detected in first larval stage worms (L1) in the intestine. From the L4 stage on through adulthood, the strongest GFP fluorescence could be detected in the intestine (A), the excretory canal cell (A) and the spermatheca (D–E). However, expression was also seen in the pharyngeal procorpus, the excretory gland cell, the pharyngeal-intestinal valve, and the rectal gland cell (A–C). During the L4 stage, the signal was also seen in vulva cells and, around the timing of the first ovulation, it is also expressed in the proximal gonad (D). Scale bar: 100 µm (A) or 10 µm (B–E).
(PDF)

Table S1 All individual defecation experiments and statistics.
(PDF)

Table S2 Measurements of total cholesterol contents and statistics.
(PDF)

Text S1 Supporting extended materials and methods.
(PDF)
