# Supplementary figures and images for: Mitochondrial Oxidative Stress Alters a Pathway in Caenorhabditis elegans Strongly Resembling That of Bile Acid Biosynthesis and Secretion in Vertebrates
Source: PLoS Genet. 2012 Mar 15;8(3):e1002553. doi: 10.1371/journal.pgen.1002553 (PMC3305355; doi:10.1371/journal.pgen.1002553)

A

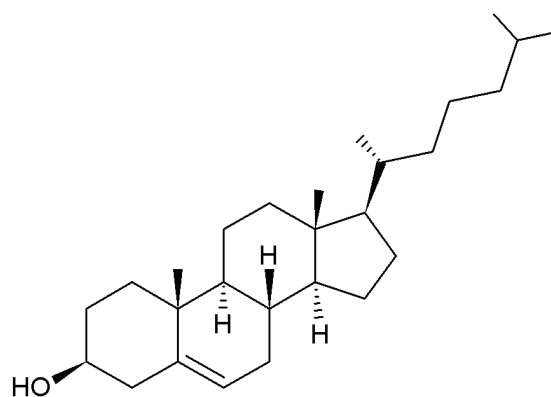

B

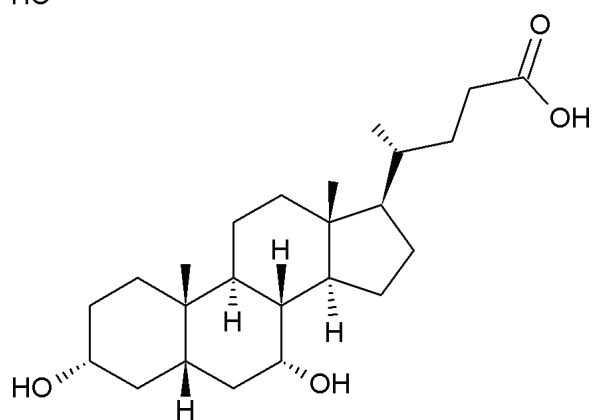

C

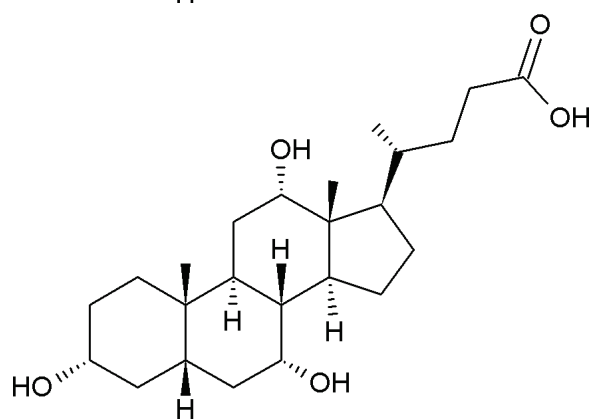

D

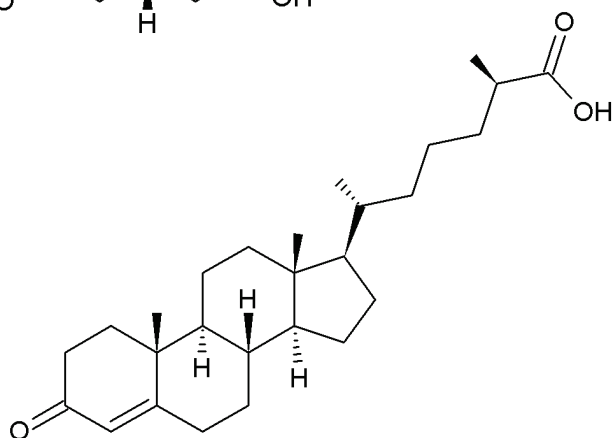

Supplement: Figure S1 — The structures of (A) cholesterol, (B) chenodeoxycholic acid (CDCA), (C) cholic acid (CA), and (D) dafachronic acid. (PDF) [file pgen.1002553.s001.pdf]

Figure S2

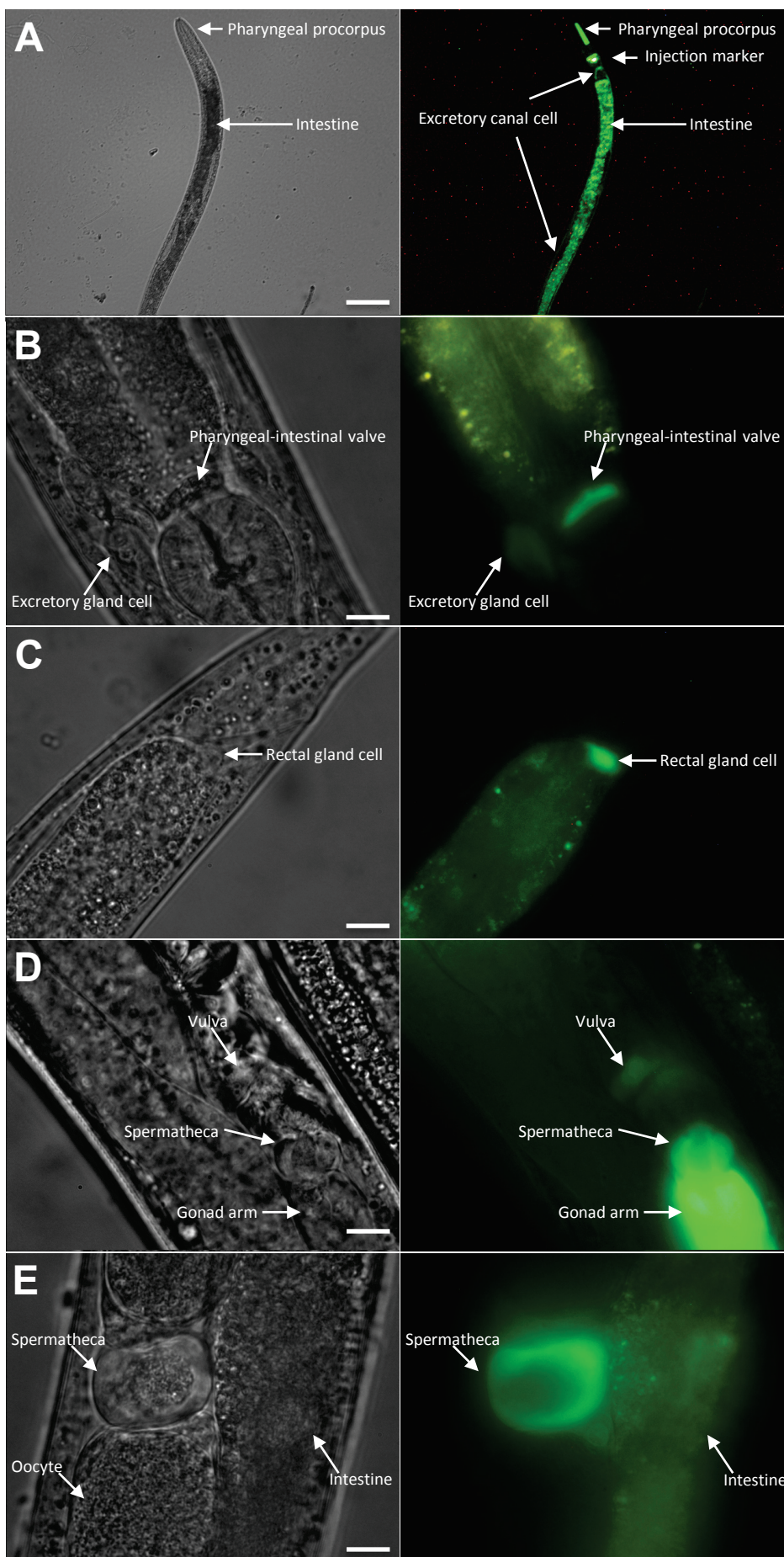

Supplement: Figure S2 — The expression pattern of the translational fusion reporter tat-2::gfp. The expression of TAT-2 was first detected in first larval stage worms (L1) in the intestine. From the L4 stage on through adulthood, the strongest GFP fluorescence could be detected in the intestine (A), the excretory canal cell (A) and the spermatheca (D–E). However, expression was also seen in the pharyngeal procorpus, the excretory gland cell, the pharyngeal-intestinal valve, and the rectal gland cell (A–C). During the L4 stage, the signal was also seen in vulva cells and, around the timing of the first ovulation, it is also expressed in the proximal gonad (D). Scale bar: 100 µm (A) or 10 µm (B–E). (PDF) [file pgen.1002553.s002.pdf]
