# Supplementary material for: Mitochondrial Oxidative Stress Alters a Pathway in Caenorhabditis elegans Strongly Resembling That of Bile Acid Biosynthesis and Secretion in Vertebrates
Source: PLoS Genet. 2012 Mar 15;8(3):e1002553. doi: 10.1371/journal.pgen.1002553 (PMC3305355; doi:10.1371/journal.pgen.1002553)
Supplement: Table S1 — All individual defecation experiments and statistics. (PDF) [file pgen.1002553.s003.pdf]

Supporting Table S1: Individual defecation experiments and statistics

Genotype	Treatment	Mean defecation cycle length (sec) Mean ± SEM	Maximum defecation cycle length (sec)	p-values vs. control*	Mean defecation cycle length change (sec)
**Wild type (N2)**	Control#	58.0±0.7 (n=28)	70.2		
dsc-4(qm182)		49.7±0.6 (n=28)	56.0	P<0.0001	-8.3
dsc-3(qm179)		51.0±0.8 (n=28)	60.4	P<0.0001	-7.0
dsc-4(qm182); dsc-3(qm179)		50.3±0.5 (n=25)	55.0	P<0.0001	-7.7
clk-1(qm30)	Control	91.3±2.6 (n=27)	131.4		
clk-1(qm30); dsc-4(qm182)		67.0±0.8 (n=27)	76.8	P<0.0001	-24.3
clk-1(qm30); dsc-3(qm179)		63.0±0.8 (n=25)	72.0	P<0.0001	-28.3
clk-1(qm30); dsc-4(qm182); dsc-3(qm179)		64.4±1.1 (n=30)	79.8	P<0.0001	-26.9
**Wild type (N2)**	Control	55.1±0.5 (n=20)	59.6		
qm179		51.7±0.5 (n=23)	56.4	P<0.0001	-3.4
tat-2(tm1634)		50.9±0.8 (n=20)	57.2	P<0.0001	-4.2
qm179/tat-2(tm1634)		51.7±0.5 (n=29)	58.2	P<0.0001	-3.4
clk-1(qm30)	Control	89.7±4.4 (n=20)	148.7		
clk-1(qm30); qm179		64.8±0.6 (n=20)	71.2	P<0.0001	-24.9
clk-1(qm30); tat-2(tm1634)		65.9±1.2 (n=23)	79.2	P<0.0001	-23.8
clk-1(qm30); qm179/tat-2(tm1634)		63.0±1.6 (n=21)	83.2	P<0.0001	-26.7
clk-1(qm30); qm179; qmEx[tat-2]		93.4±3.3 (n=20)	128.4		
**Wild type (N2)**	Empty vector (HT115) (Control)	61.0±0.6 (n=27)	67.4		
	tat-2 RNAi	58.1±0.5 (n=29)	62.2	P=0.0001	-2.9

clk-1(qm30)	Empty vector (HT115) (Control)	91.5±3.1 (n=29)	136.3		
	tat-2 RNAi	71.3±1.3 (n=33)	88.3	P<0.0001	-20.2
clk-1(qm30); tat-2(qm179)	Empty vector (HT115) (Control)	65.5±2.0 (n=20)	79.8		
clk-1(qm30); tat-2(qm179)	*mAtp8b1* RNAi	67.0±2.3 (n=20)	81.0		
clk-1(qm30); tat-2(qm179); qmEx[Ptat-2::mAtp8b1]	Empty vector (HT115)	80.6±6.5 (n=20)	166.0	P=0.032	+15.1
clk-1(qm30); tat-2(qm179); qmEx[Ptat-2::mAtp8b1]	*mAtp8b1* RNAi	66.2±2.8 (n=21)	103.4		
clk-1(qm30); tat-2(qm179)	Control	65.7±0.7 (n=21)	71.2		
clk-1(qm30); tat-2(qm179); qmEx[Ptat-2::mAtp8b1]		75.9±3.0 (n=20)	118.2	P=0.0017	+10.2
clk-1(qm30); tat-2(qm179); qmEx[Ptat-2::mAtp8b1 A705T]		65.6±1.2 (n=20)	74.4		
clk-1(qm30); tat-2(qm179); qmEx[Ptat-2::mAtp8b1 G308V]		64.0±0.9 (n=20)	72.4		
clk-1(qm30); tat-2(qm179)	Control	65.6±1.5 (n=20)	78.4		
clk-1(qm30); tat-2(qm179); qmEx[Ptat-2::tat-2::gfp]		85.0±4.7 (n=20)	133.4	P=0.0003	+19.4
clk-1(qm30); tat-2(qm179); qmEx[Pges-1::tat-2::gfp]		82.5±3.7 (n=32)	161.2	P=0.0010	+16.9
clk-1(qm30); tat-2(qm179); qmEx[Psth-1::tat-2::gfp]		64.3±1.7 (n=20)	77.4		
clk-1(qm30); tat-2(qm179);		66.8±1.3 (n=20)	76.0		

qmEx[Ppgp-12::tat-2::gfp]					
**Wild type (N2)**	Control	55.9±0.5 (n=54)	66.2		
	0.025% Cholestyramine	55.6±0.4 (n=50)	66.0		
clk-1(qm30)	Control	95.1±2.3 (n=50)	137.0		
	0.025% Cholestyramine	84.4±1.6 (n=66)	131.7	P=0.0001	-10.7
isp-1(qm150)	Control	104.3±2.4 (n=53)	161.3		
	0.025% Cholestyramine	102.8±1.8 (n=64)	149.0		
tat-2(qm179)	Control	51.9±0.3 (n=50)	56.6		
	0.025% Cholestyramine	51.4±0.4 (n=50)	57.2		
dsc-4(qm182)	Control	49.7±0.3 (n=51)	55.4		
	0.025% Cholestyramine	49.0±0.4 (n=51)	55.6		
clk-1(qm30); tat-2(qm179)	Control	63.4±0.9 (n=50)	90.4		
	0.025% Cholestyramine	63.1±0.7 (n=50)	74.4		
clk-1(qm30); dsc-4(qm182)	Control	66.6±0.6 (n=51)	80.2		
	0.025% Cholestyramine	68.0±0.6 (n=50)	77.4		
**Wild type (N2)**	Control	58.4±0.4 (n=24)	61.6		
daf-36(k114)		56.9±0.4 (n=25)	62.8	P=0.0122	-1.5
clk-1(qm30)	Control	95.4±2.1 (n=26)	125.3		
clk-1(qm30); daf-36(k114)		76.0±1.6 (n=27)	107.7	P<0.0001	-19.4
**Wild type (N2)**	Control	55.6±0.4 (n=26)	63.0		
	0.015% Bile	55.5±0.4 (n=20)	59.0		
clk-1(qm30)	Control	87.3±2.8 (n=20)	113.2		
	0.015% Bile	115.2±5.6 (n=20)	172.3	P<0.0001	+27.9

dsc-4(qm182)	Control	49.3±0.3 (n=20)	52.0		
	0.015% Bile	50.2±0.5 (n=21)	54.4		
tat-2(qm179)	Control	50.8±0.6 (n=20)	55.4		
	0.015% Bile	56.3±0.8 (n=20)	66.6	P<0.0001	+5.5
	Cholesterol free+ 0.015% Bile	52.0±0.8 (n=23)	61.6		
clk-1(qm30); tat-2(qm179)	Control	63.9±1.1 (n=20)	75.2		
	0.015% Bile	86.0±1.8 (n=20)	103.4	P<0.0001	+22.1
	Cholesterol free+ 0.015% Bile	63.1±1.3 (n=21)	72.6		
**Wild type (N2)**	0.25% DMSO (Control)	59.6±1.1 (n=23)	72.2		
	0.25% DMSO+ 0.15mM cholic acid	59.0±0.7 (n=20)	65.2		
	0.25% DMSO+ 0.6mM cholic acid	60.2±0.5 (n=25)	67.2		
	0.25% DMSO+ 2.5mM cholic acid	59.4±1.0 (n=20)	64.6		
	0.25% DMSO+ 0.15mM CDCA	59.8±0.7 (n=22)	64.4		
	0.25% DMSO+ 0.6mM CDCA	59.0±0.9 (n=21)	68.8		
	0.25% DMSO+ 2.5mM CDCA	58.2±0.9 (n=21)	68.6		
clk-1(qm30)	0.25% DMSO (Control)	105.3±2.5 (n=77)	174.3		
	0.25% DMSO+ 0.15mM cholic acid	90.7±2.6 (n=40)	138.3	P=0.0003	-14.6
	0.25% DMSO+ 0.6mM cholic acid	96.8±2.0 (n=58)	130.7	P=0.0114	-8.5
	0.25% DMSO+ 2.5mM cholic acid	144.0±4.4 (n=40)	227.7	P<0.0001	+38.7
	0.25% DMSO+ 0.15mM CDCA	103.3±3.6 (n=41)	175.7		
	0.25% DMSO+ 0.6mM CDCA	119.6±4.1 (n=43)	186.7	P=0.0020	+14.3
	0.25% DMSO+ 2.5mM CDCA	136.7±5.0 (n=40)	239.3	P<0.0001	+31.4
clk-1(qm30)	Cholesterol free+ 0.25% DMSO (Control)	78.2±2.4 (n=41)	127.7		

	Cholesterol free+ 0.25% DMSO + 0.15mM cholic acid	82.1±2.1 (n=41)	115.3		
	Cholesterol free+ 0.25% DMSO + 0.6mM cholic acid	81.5±2.3 (n=40)	139.7		
	Cholesterol free+ 0.25% DMSO + 2.5mM cholic acid	83.4±1.4 (n=42)	109.3		
	Cholesterol free+ 0.25% DMSO + 0.15mM CDCA	79.0±2.1 (n=41)	120.3		
	Cholesterol free+ 0.25% DMSO + 0.6mM CDCA	81.7±1.9 (n=43)	118.3		
	Cholesterol free+ 0.25% DMSO + 2.5mM CDCA	99.0±2.4 (n=42)	129.3	P<0.0001	+20.8
**Wild type (N2)**	0.25% DMSO (Control)	59.4±0.6 (n=28)	66.6		
	0.25% DMSO+ 0.02mg N2 lipid extract	58.5±0.6 (n=29)	67.6		
	0.25% DMSO+ 0.1mg N2 lipid extract	60.2±0.4 (n=30)	64.8		
	0.25% DMSO+ 0.4mg N2 lipid extract	58.7±0.3 (n=25)	61.6		
	0.25% DMSO+ 0.02mg *clk-1* lipid extract	58.2±0.7 (n=26)	65.2		
	0.25% DMSO+ 0.1mg *clk-1* lipid extract	58.4±0.8 (n=28)	69.0		
clk-1(qm30)	0.25% DMSO (Control)	97.0±1.8 (n=25)	115.7		
	0.25% DMSO+ 0.02mg N2 lipid extract	98.8±3.3 (n=28)	152.0		
	0.25% DMSO+ 0.1mg N2 lipid extract	102.0±2.5 (n=25)	123.3		
	0.25% DMSO+ 0.4mg N2 lipid extract	116.7±2.5 (n=25)	139.0	P<0.0001	+19.7
	0.25% DMSO+ 0.02mg *clk-1* lipid extract	108.3±3.7 (n=28)	169.0	P=0.0114	+11.3

	0.25% DMSO+ 0.1mg *clk-1* lipid extract	114.8±3.5 (n=26)	149.7	P<0.0001	+17.8
**Wild type (N2)**	Control (5ug/ml cholesterol)	55.2±0.5 (n=28)	64.2		
	Low Cholesterol (2ug/ml)	54.9±0.8 (n=25)	63.2		
	High Cholesterol (50ug/ml)	55.0±0.6 (n=29)	62.0		
clk-1(qm30)	Control	92.7±1.8 (n=25)	109.0		
	Low Cholesterol	72.9±1.6 (n=26)	102.0	P<0.0001	-19.8
	High Cholesterol	112.3±2.5 (n=28)	148.0	P<0.0001	+19.6
clk-1(qm30); tat-2(qm179)	Control	65.9±1.0 (n=25)	78.2		
	Low Cholesterol	60.4±0.7 (n=25)	65.8	P<0.0001	-5.5
	High Cholesterol	65.9±1.2 (n=26)	79.6		
tat-2(qm179)	Control	51.6±0.5 (n=26)	56.2		
	Low Cholesterol	49.9±0.5 (n=25)	53.6	P=0.0168	-1.7
	High Cholesterol	52.8±0.6 (n=25)	57.0		
***clk-1(qm30)* (25°C)**	Empty vector (HT115) (Control)	85.0±2.2 (n=41)	127		
	sod-1 RNAi	92.8±4.1 (n=6)	111		
	sod-2 RNAi	115.8±5.2 (n=12)	151	P<0.0001	+30.8
	sod-3 RNAi	98.3±2.7 (n=24)	127	P=0.0004	+13.3
	sod-4 RNAi	87.6±2.7 (n=29)	120		
	sod-5 RNAi	85.7±2.9 (n=24)	115		
**Wild type (N2) (25°C)**	Control	41.3±1.6 (n=6)	45		
	2mM NAC	43.2±0.5 (n=14)	47		
	4mM NAC	42.3±0.7 (n=14)	46		
	6mM NAC	39.0±1.2 (n=7)	42		

	8mM NAC	43.3±0.6 (n=15)	48		
	10mM NAC	41.9±0.9 (n=7)	44		
***clk-1(qm30)* (25°C)**	Control	82.5±2.7 (n=27)	112		
	2mM NAC	81.7±3.5 (n=23)	121		
	4mM NAC	81.6±2.8 (n=22)	107		
	6mM NAC	78.0±2.4 (n=23)	101		
	8mM NAC	74.9±2.2 (n=25)	99	P=0.0348	-7.6
	10mM NAC	69.2±2.2 (n=24)	91	P=0.0004	-13.3
**Wild type (N2)**	Control	53.3±0.9 (n=15)	58		
	10mM NAC	51.6±1.0 (n=15)	60		
clk-1(qm30)	Control	73.9±2.2 (n=15)	93		
	10mM NAC	64.1±1.3 (n=13)	72	P=0.001	-9.8
clk-1(qm30); tat-2(qm179)	Control	50.3±2.9 (n=6)	58		
	10mM NAC	50.9±2.1 (n=7)	59		
**Wild type (N2)**	0.25% DMSO (Control)	58.6±0.4 (n=26)	64.6		
	0.25% DMSO + 0.1mg N2 lipid extract	59.2±0.7 (n=26)	66.4		
	0.25% DMSO + 0.1mg *clk-1* lipid extract	58.5±0.4 (n=25)	63.6		
	0.25% DMSO + 0.1mg *clk-1;* NAC lipid extract	58.5±0.7 (n=25)	64.4		
	0.25% DMSO + 0.1mg *clk-1; sod-2* lipid extract	58.5±0.5 (n=26)	65.6		

clk-1(qm30)	0.25% DMSO (Control)	93.5±3.0 (n=26)	126.3		
	0.25% DMSO<br>+ 0.1mg N2 lipid extract	106.8±3.0 (n=27)	149.0	P=0.0026	+13.3
	0.25% DMSO<br>+ 0.1mg *clk-1* lipid extract	122.5±5.0 (n=25)	180.0	P<0.0001	+29.0
	0.25% DMSO<br>+ 0.1mg *clk-1;* NAC lipid extract	106.4±2.8 (n=28)	153.7	P=0.0026	+12.9
	0.25% DMSO<br>+ 0.1mg *clk-1; sod-2* lipid extract	160.3±26.0 (n=30)	848.0	P=0.0210	+66.8

*When experiments have been carried out more than once, the p-values are for comparisons between pooled datasets for each condition.

Unless different levels of cholesterol supplementation, the use of the RNAi strain HT115, or scoring at 25°C is specified, all experiments were carried out at 20°C with OP50 and the standard 5μg/ml of cholesterol.

.
