# Supplementary material for: Mitochondrial Oxidative Stress Alters a Pathway in Caenorhabditis elegans Strongly Resembling That of Bile Acid Biosynthesis and Secretion in Vertebrates
Source: PLoS Genet. 2012 Mar 15;8(3):e1002553. doi: 10.1371/journal.pgen.1002553 (PMC3305355; doi:10.1371/journal.pgen.1002553)
Supplement: Table S2 — Measurements of total cholesterol contents and statistics. (PDF) [file pgen.1002553.s004.pdf]

Supporting Table S2: Measurements of total cholesterol contents and statistics

Treatment	Genotype	Cholesterol level Mean ± SEM	Maximum cholesterol level	p-values vs. control	Mean cholesterol level change
**Regular NGM (5µg/ml cholesterol)**	Wild type (N2) (Control)	1.000 ±0.019 (n=19)[#]	1.190		
	clk-1(qm30)	1.057 ±0.046 (n=18)	1.681		
	tat-2(qm179)	1.098 ±0.021 (n=16)	1.319	P=0.0015	+0.098
	clk-1(qm30); tat-2(qm179)	1.230 ±0.032 (n=16)	1.559	P<0.0001	+0.230
**Low cholesterol (2µg/ml)**	Wild type (N2) (Control)	1.042 ±0.041(n=16)	1.302		
	clk-1(qm30)	1.061 ±0.031 (n=16)	1.359		
	tat-2(qm179)	0.987 ±0.018 (n=16)	1.097		
	clk-1(qm30); tat-2(qm179)	1.311 ±0.029 (n=16)	1.654	P<0.0001	+0.269
**High cholesterol (50µg/ml)**	Wild type (N2) (Control)	1.349 ±0.047(n=16)	1.660		
	clk-1(qm30)	1.601 ±0.049 (n=16)	1.937	P=0.0008	+0.252
	tat-2(qm179)	1.319 ±0.047 (n=16)	1.655		
	clk-1(qm30); tat-2(qm179)	1.314 ±0.014 (n=16)	1.401		

[#] All cholesterol levels were normalized to the wild type at 5µg/ml of cholesterol supplementation. This wild type concentration was 0.056µM.
