# Supplementary material for: Mitochondrial Oxidative Stress Alters a Pathway in Caenorhabditis elegans Strongly Resembling That of Bile Acid Biosynthesis and Secretion in Vertebrates
Source: PLoS Genet. 2012 Mar 15;8(3):e1002553. doi: 10.1371/journal.pgen.1002553 (PMC3305355; doi:10.1371/journal.pgen.1002553)
Supplement: Text S1 — Supporting extended materials and methods. (PDF) [file pgen.1002553.s005.pdf]

Text S1: Supporting extended Materials and Methods

General methods and strains

Most strains were derived from the wild type *C. elegans* N2 Bristol strain and were cultured as described (Brenner 1974). All animals were grown at 20°C. Unless otherwise indicated, the worms were cultured on regular NGM plates supplemented with the standard amount of cholesterol (5µg/ml). High cholesterol plates were supplemented with cholesterol at a concentration of 50µg/ml; low cholesterol plates were supplemented with 2µg/ml cholesterol; cholesterol-free plates were not supplemented with media cholesterol. In the latter, trace amounts of cholesterol are likely present from other components of the growth media. For testing the effect of cholestyramine (C4650), mixed bile acids (B8381), cholic acid (C1129), chenodeoxycholic acid (22510), and N-acetyl-L-cysteine (A7250) (all obtained from Sigma-Aldrich), compounds were dissolved in the appropriate solvents, and spread onto plates, except N-acetyl-L-cysteine, which was added to the NGM prior to pouring media into plates. The final concentrations of cholestyramine, mixed bile acids, cholic acid, chenodeoxycholic acid and N-acetyl-L-cysteine are described in the results. Fourth larval stage (L4) animals were transferred to the test plates and grown at 20°C. The effects of the different cholesterol concentrations or compounds were scored after raising the worms on the test plates for one generation. Defecation rates were measured as previously described (Branicky, Shibata et al. 2001), at 20°C for most experiments except for the RNAi and antioxidant treatments for which 25°C was used. Student's *t*-test was used to determine statistical significance and was performed using Graphpad Prism version 5.0 software.

The *dsc-3(qm179)* and *dsc-4(qm182)* alleles were isolated in an EMS mutagenesis screen (Branicky, Shibata et al. 2001) and outcrossed three times; the *tat-2(tm1634)* allele was obtained from the National Bioresource Project and outcrossed three times. The mutations used in this study were as follows: *clk-1(qm30)*, *isp-1(qm150)*, *dsc-4(qm182)*, *dsc-3(qm179), tat-2(tm1634), unc-33(e204), dpy-4(e166), unc-5(e53)*, *dpy-13(e184), sod-2(ok1030) and daf-36(k114)*.

Positional cloning of *dsc-3(qm179)*

dsc-3 had previously been mapped to LG IV, between *unc-33* and *dpy-4* (Branicky, Shibata et al. 2001). By using 2-point and 3-point mapping strategies, the genetic position of *qm179* was refined to the position between the two cloned gene *dpy-13* and *unc-5*. Due to the incomplete cosmid coverage of the *tat-2* gene, no cosmid that spans this region can rescue the *qm179* mutants. Therefore *qm179*mutants were rescued by injecting two partially overlapping PCR fragments of *tat-2* genomic DNA (from -3123 to +7277 and from +7252 to +13567, which includes the UTRs) for in vivo recombination (Kemp, Hatzold et al. 2007). The two PCR products were injected at a concentration of

40 ng/µl each with the co-injection marker *inx-6::gfp* at a concentration of 180 ng/µl. To determine the lesion of the *qm179* mutation, genomic DNA was extracted from *qm179*mutants and the predicted coding region was sequenced. A G-to-A transition was identified at position 1993 of the coding region relative to the ATG resulting in an A-to-T substitution at residue 665 of the TAT-2 protein.

Construction of plasmids and transgenic strains

The *tat-2* transcriptional reporter, *Ptat-2::gfp* (pCDB898) was used as backbone for subcloning. The PCR product of 3.4 kb upstream of the initiating ATG of *tat-2* was amplified from N2 genomic DNA and was cloned into the PstI and SmaI sites of the pPD95_77 vector, kindly provided by A. Fire. All the following constructs were injected into *clk-1; tat-2(qm179)* mutants. Multiple transgenic lines were obtained in each experiment.

Construction of pCDB908, pCDB901 and pCDB900 (*Ptat-2::mAtp8b1, Ptat-2::mAtp8b1 A705T, Ptat-2::mAtp8b1 G308V,* respectively): To construct these clones the full length of mouse *Atp8b1* cDNA was amplified from the RIKEN clone F830210O18 which was obtained through DNAFORM. PCR product was cloned into the SmaI and XhoI sites of the pCDB898. pCDB901 and pCDB900 were generated by site-directed mutagenesis from pCDB908 as the template and the mutations were sequenced (Fisher and Pei 1997). Construct of pCDB908, pCDB901 or pCDB900 was injected at a concentration of 1ng/µl along with the transformation marker *inx-6::gfp* at a concentration of 199ng/µl. The accession number for the mouse RIKEN clone F830210O18 is AK157316.

Construction of pCDB902 (*Ptat-2::tat-2::gfp)* was carried by the reverse transcription of total RNA extracted from wild-type worms and the *tat-2* transcript was amplified with primers corresponding to the predicted transcript. The *tat-2* cDNA contains 22 exons and is 3945 bp long. The PCR product excluding the stop codon was inserted into the SmaI site of pCDB898.

Construction of pCDB906, pCDB905 and pCDB904 (*Pges-1::tat-2::gfp*, *Psth-1::tat-2::gfp, Ppgp-12::tat-2::gfp*, respectively): The *tat-2* promoter of pCDB902 was replaced by PCR products of 2kb upstream of the *ges-1* initiation codon (Aamodt, Chung et al. 1991), 1.6kb upstream of the *sth-1* initiation codon (Bando, Ikeda et al. 2005) or 2.7 kb upstream of the *pgp-12* initiation codon (Zhao, Fang et al. 2005). The pCDB902, pCDB906, pCDB905 or pCDB904 constructs were injected at a concentration of 0.1ng/µl along with the transformation marker *ttx-3::gfp* at a concentration of 200ng/µl.

Biochemical measurement of total cholesterol content

To measure the cholesterol content of the wild type, *clk-1(qm30)*, *tat-2(qm179)* and *clk-1(qm30); tat-2(qm179)* mutants under different levels of cholesterol supplementation, L4

worms grown on regular NGM plates in the presence of 5µg/ml of cholesterol were transferred to plates containing 2µg/ml, 5µg/ml or 50µg/ml cholesterol and 1000 young adult progeny were collected into one Eppendorf tube as one sample. Worms were washed with water to remove bacteria and were freshly frozen in liquid nitrogen and stored at -80°C until further processing. As previously described (Brock, Browse et al. 2006), lipids were extracted by mixing and then incubating with ice cold chloroform: methanol (1:1) overnight at -20°C. The samples were washed with Hajra's solution(0.2M H_3P0_4, 1M KCl) and the lower chloroform phase containing lipids was collected. To re-extract, additional chloroform was added to samples. The combined chloroform extracts were evaporated and the sides of Eppendorf tube were washed by chloroform containing 0.5% Triton X-100. The solvent in each tube was evaporated and lipids were resuspended in water for 15 minutes at 37°C. The final concentration of Triton X-100 in each sample was 0.5%. The total cholesterol contents were determined using an enzymatic kit (10007640; Cayman Chemical) according to the manufacturer's protocol. We also measured the volumes of young adults for all genotypes as previously described [40], and no difference from the wild type was found (data not shown).

Active lipid extracts

To get a highly synchronous population of first larval stage (L1) animals, 120x 5cm NGM plates of gravid worms were bleached to obtain embryos which were allowed to hatch overnight in M9 buffer. Nematodes were then transferred to 120x 9cm NGM plates which are supplied with or without NAC at the concentration of 10mM and covered with *E. coli* OP50, allowed to grow to the young adult stage. Worms were washed three times with water before the worm pellet was frozen in liquid nitrogen and stored at -80°C. The lipid extracts were then prepared as previously described (Gill, Held et al. 2004) and the resulting lipids were weighed and resuspended in DMSO. To assay the activity of extracts from the wild type, *clk-1(qm30), clk-1(qm30); sod-2(ok1030)* or *clk-1(qm30)* mutants treated with NAC, 36µl of DMSO-dissolved extract (or 36µl of DMSO as control) was spread onto 5cm plates and the effects of the activity were measured by scoring defecation phenotype of adult progeny after raising L4 animals on the test plates for one generation. Due to the sensitivity of *clk-1* mutants to dietary cholesterol level, we measured and calculated that the final concentrations of extracts applied to the plates contained less than 0.1µg/ml of cholesterol, which cannot therefore be responsible for any of the effects observed (Figure 2D).

RNAi feeding

RNAi feeding was conducted as previously described (Kamath, Martinez-Campos et al. 2001). To study whether ATP8B1 and TAT-2 carry out the same function a portion of the mouse *Atp8b1* cDNA (from 1510 bp to 1933 bp of the coding region relative to the initiation ATG) was amplified from the RIKEN clone F830210O18 and was cloned into

the EcoRV and HindIII sites of the pPD129.36 vector, kindly provided by A. Fire. *tat-2* RNAi was obtained from the *C. elegans* RNAi library (Geneservice). Fourth larval stage (L4) animals were transferred to the RNAi plates and grown at 20°C. Defecation cycle rates were scored for analyzing the effect of the mouse *Atp8b1* RNAi or *tat-2* RNAi in P0 or F1 generation, respectively. Transformed HT115 with the empty pPD129.36 vector were used as control.

To identify *clk-1* suppressors 5-10 *clk-1(qm30)* hermaphrodites L4 larvae were picked to RNAi plates. For the following 3 days, worms were transferred to new RNAi plates to get rid of contaminating OP50 bacteria. Progeny worms were grown to the L4 stage and were then picked to new RNAi plates for scoring. 18 hours later, they were transferred to 25°C. After two hours of acclimation, their defecation phenotype was scored. We used 25°C for RNAi screen because the responses tend to be more robust (Branicky, Shibata et al. 2001). For each RNAi clone, five worms were scored for one defecation cycle. Clones that had a significant effect on defecation rate were re-screened 2-3 times.

Aamodt, E. J., M. A. Chung, et al. (1991). "Spatial control of gut-specific gene expression during Caenorhabditis elegans development." Science **252**(5005): 579-82.

Bando, T., T. Ikeda, et al. (2005). "The homeoproteins MAB-18 and CEH-14 insulate the dauer collagen gene col-43 from activation by the adjacent promoter of the Spermatheca gene sth-1 in Caenorhabditis elegans." J Mol Biol **348**(1): 101-12.

Brenner, S. (1974). "The genetics of Caenorhabditis elegans." Genetics **77**(1): 71-94.

Fisher, C. L. and G. K. Pei (1997). "Modification of a PCR-based site-directed mutagenesis method." Biotechniques **23**(4): 570-1, 574.

Kamath, R. S., M. Martinez-Campos, et al. (2001). "Effectiveness of specific RNA-mediated interference through ingested double-stranded RNA in Caenorhabditis elegans." Genome Biol **2**(1): RESEARCH0002.

Kemp, B. J., J. Hatzold, et al. (2007). "In vivo construction of recombinant molecules within the Caenorhabditis elegans germ line using short regions of terminal homology." Nucleic Acids Res **35**(19): e133.

Zhao, Z., L. Fang, et al. (2005). "Distinct regulatory elements mediate similar expression patterns in the excretory cell of Caenorhabditis elegans." J Biol Chem **280**(46): 38787-94.
